# Identification and Evaluation of Inhibitors of Lipase from *Malassezia restricta* using Virtual High-Throughput Screening and Molecular Dynamics Studies

**DOI:** 10.3390/ijms20040884

**Published:** 2019-02-18

**Authors:** Shahid Ali, Faez Iqbal Khan, Taj Mohammad, Dongming Lan, Md. Imtaiyaz Hassan, Yonghua Wang

**Affiliations:** 1 School of Food Science and Engineering, South China University of Technology, Guangzhou 510640, China; ali.ali.md111@gmail.com (S.A.); dmlan@scut.edu.cn (D.L.); 2 Department of Chemistry, Rhodes University, Grahamstown 6140, South Africa; khanfaeziqbal@gmail.com; 3 Centre for Interdisciplinary Research in Basic Sciences, Jamia Millia Islamia, New Delhi-110025, India; taj144796@st.jmi.ac.in (T.M.); imtiyaz.hassan@gmail.com (M.I.H.)

**Keywords:** traditional Chinese medicine, lipase, *Malassezia restricta*, molecular dynamics simulation, anti-dandruff, Zinc database, drug design and discovery

## Abstract

Recent studies revealed the role of lipase in the pathogenicity of *Malassezia restricta* in dandruff and seborrheic dermatitis (D/SD). The lipase from *M. restricta* (Mrlip1) is considered a potential target for dandruff therapy. In this work, we performed structure-based virtual screening in Zinc database to find the natural bioactive inhibitors of Mrlip1. We identified three compounds bearing superior affinity and specificity from the Traditional Chinese Medicine database (~60,000 compounds), and their binding patterns with Mrlip1 were analyzed in detail. Additionally, we performed three sets of 100 ns MD simulations of each complex in order to understand the interaction mechanism of Mrlip1 with known inhibitor RHC80267 and the newly identified compounds such as ZINC85530919, ZINC95914464 and ZINC85530320, respectively. These compounds bind to the active site cavity and cause conformational changes in Mrlip1. The Molecular Mechanics Poisson-Boltzmann Surface Area (MMPBSA) studies suggested that the average binding energy was stronger in the case of Mrlip1-ZINC85530919 and Mrlip1-ZINC95914464. The selected natural inhibitors might act as promising lead drugs against Mrlip1. Further, the present study will contribute to various steps involved in developing and creating potent drugs for several skin diseases including dandruff.

## 1. Introduction

Lipases (E.C. 3.1.1.3) belong to the hydrolase family that act on carboxylic ester bonds [1]. Monoacylglycerol (MAG) and diacylglycerol (DAG) lipases have been taken in consideration because of their physiological functions as a biocatalyst [2] and applications in oil and fat modification [3]. Lipases are one of the most widely used classes of enzymes in biotechnology and synthetic organic chemistry [4]. In spite of the fact that *Malassezia restricta* lipase (Mrlip1) is a mono- and diacylglycerol lipase secreted from *M. restricta KCTC27527*, it was isolated and identified from a patient with dandruff and seborrheic dermatitis (D/SD) [5,6]. Dandruff is a common scalp disorder that has a prevalence of nearly 50% in the worldwide population [7]. Several studies have claimed associations between *M. restricta* and specific skin diseases [8]. The *Malassezia spp.* lipases degrade the triglycerides of human sebum and consume specific saturated fatty acids which in turn cause irritation in individuals’ skin with dandruff and seborrhoeic dermatitis [9].

It has been well established that pathogenic fungi produce extracellular lipases to breach the host tissue barrier and enable them to penetrate the tissue. The examples include lipases of *Candida albicans* that contribute to yeast-hypha transition, colonization and invasion into the host tissues [10,11]. Moreover, the lipases were also identified in *M. furfur* and *M. globosa*, and their biochemical characteristics were analyzed [9,12].

Current treatments of D/SD are mostly anti-fungal agents such as zinc pyrithione, ketoconazole, coal tar, selenium sulfide, and general lipase inhibitors [13]. However, efficient, stable and non-polymeric therapeutic agents are much needed. The activity of Mrlip1 is likely to be maximal under the conditions that *M. restricta* typically faces at the host skin surface and involved in dandruff diseases [6].

The present work aims to seek a new class of inhibitors specifically targeting the Mrlip1 by Virtual High-throughput Screening (vHTS) and molecular dynamics (MD) simulation methods. We have performed structure-based vHTS of about 60,000 small compounds in Traditional Chinese Medicines (TCM)-based naturally occurring compounds from non-commercial ZINC database followed by several sets of 100 *ns* all-atom MD simulations to find out potent inhibitors of Mrlip1. The results suggested that ZINC95914464 is a potent bioactive compound that represents novel hits that could serve as the starting point for the development of more potent anti-dandruff therapeutic agents.

## 2. Results and Discussion

The *Malassezia restricta* lipase (Mrlip1) is mainly involved in dandruff progression [6]. Amongst all species of dandruff causing *Malassezia*, the most dominated species is *M. restricta* [7]. Therefore, a total of about 60,000 small compounds were screened against the structure of Mrlip1, and 80 top-ranked compounds that have the highest binding affinity were selected for further screening. All novel hits were accurately fitted within the active site of Mrlip1 and were further evaluated for drug-likeness using various tools.

### 2.1. vHTS: Molecular Docking

The screening of compounds library produced log-files and output-files, which contain binding affinity scores and docked poses for individual compounds in the library. These log-files and output-files were subjected to a screen-out based on their binding affinities, docking score and binding orientation for Mrlip1. The number of natural compounds having a good binding affinity score were selected further in the search for potential inhibitors of Mrlip1.

### 2.2. Hit Selection and Drug-Ability Assessment

Initially, the compounds were filtered out to get the highest binding affinity natural compounds from the 60,000 screened-compounds, which were extracted by a python script. We obtained the 80 highest binding affinity natural compounds (Table S1). Further, these compounds were subjected to further screening based on their physicochemical properties, where 25 compounds were qualified in specific cut-off values of drug-likeliness. The compounds were selected based on parameters such as hydrogen bond donors less than 5, hydrogen bond accepters less than 10, rotatable bonds less than 10, molecular weight less than 750 Dalton, and logP less than 10 (Table 1).

However, some compounds violated the Lipinski rule of five like many FDA approved drugs do, as they have a molecular weight of >500 Dalton and logP value >5, but this is acceptable [14]. ADMET properties of these compounds were predicted, where 14 compounds were showing acceptable ADMET properties (Table 2). All these results indicate that the selected compounds show an ideal characteristic behaviour of a drug-like molecule. Furthermore, interaction analysis was carried out to get selective compounds specific to the binding site of Mrlip1, and finally, the three best compounds were selected and one additional reported compound was taken as a control inhibitor RHC80267 (Table 3). These selected compounds passed the Pan-assay interference compounds (PAINS) filters, where no alert of PAINS pattern was found for these compounds, which supports the specificity of the selected compounds towards Mrlip1. Based on our results, we proposed that these three drug-like compounds may be considered as potent inhibitors of Mrlip1, showing appreciable binding affinity and specificity to the Mrlip1 binding pocket, which can reduce the accessibility of the substrate and thus, interfere with enzyme activity.

### 2.3. Structural Analysis of Mrlip1 Complexes

**Figure 1:**
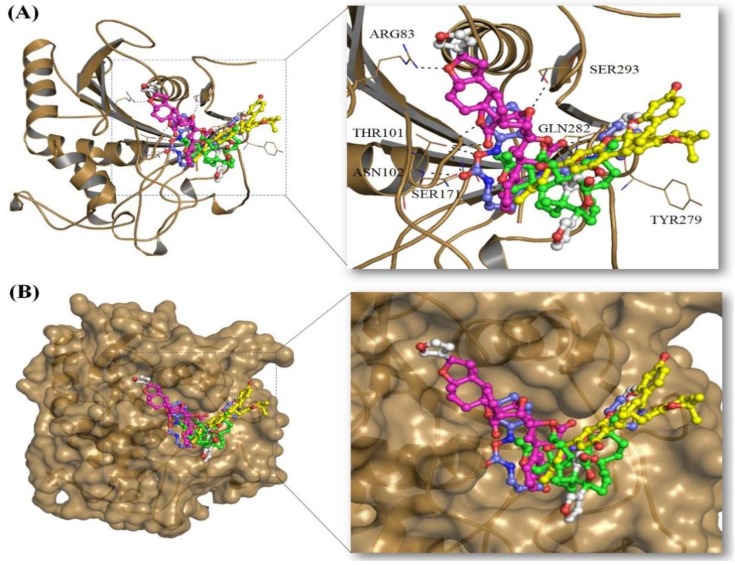
Binding mode of selected compounds: (**A**) Cartoon representation of Mrlip1 complexed with all four compounds; (**B**) surface representation of Mrlip1 complexed with all four compounds (compounds shown in ball and stick models; RHC80267 (Blue element), ZINC85530919 (Yellow element), ZINC85530320 (Green element) and ZINC95914464 (Magenta element)).

**Figure 2:**
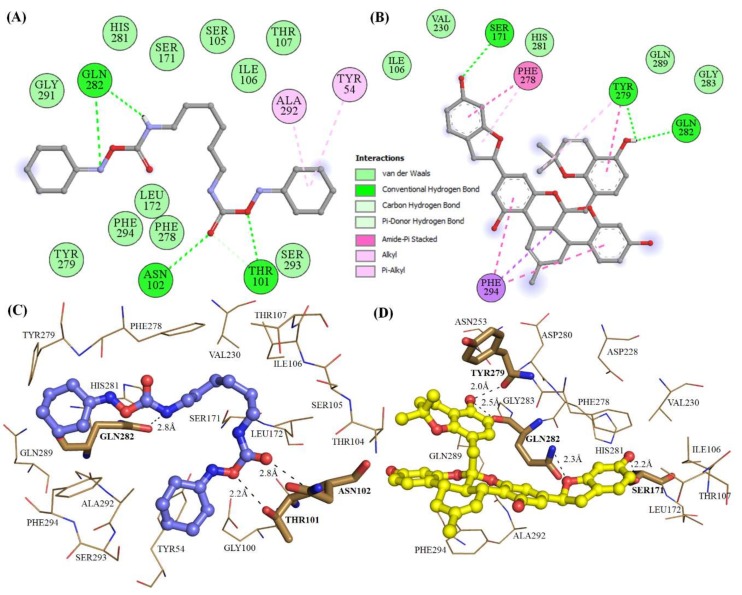
Catalytic pocket’s residue interactions of Mrlip1 with compounds. (**A**–**B**) 2D diagram of Mrlip1 residues interacting with compound RHC80267 and ZINC85530919 respectively. (**C**–**D**) 3D view of binding pocket residues of Mrlip1 interacting with compound RHC80267 and ZINC85530919 respectively. (Bold-labeled residues in stick model: forming polar contacts with the ligands and residues showing in lines participating in other interactions).

**Figure 3:**
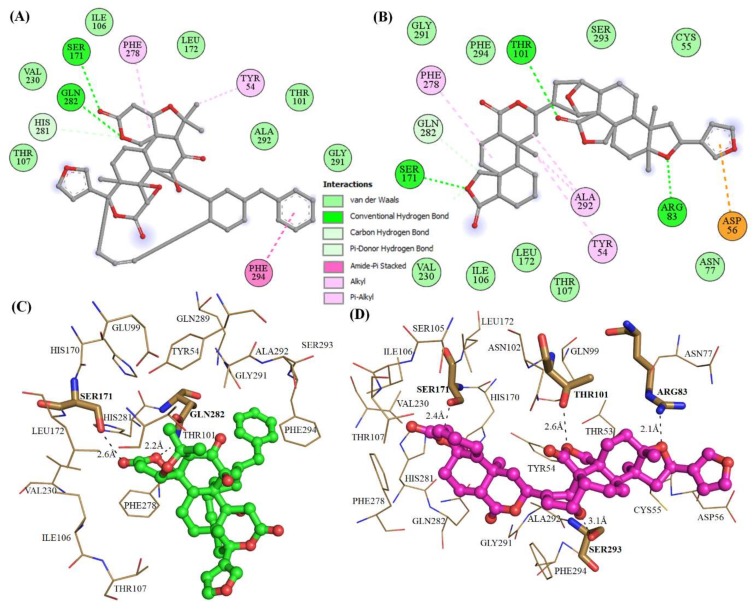
Catalytic pocket’s residues interactions of Mrlip1 with compounds. (**A**–**B**) 2D diagram of Mrlip1 residues interacting with compound ZINC85530320 and ZINC95914464 respectively. (**C**–**D**) 3D view of binding pocket residues of Mrlip1 interacting with compound ZINC85530320 and ZINC95914464 respectively. (Bold-labeled residues in stick model: forming polar contacts with the ligands and residues showing in lines participating in other interactions).

The structural analysis of Mrlip1 complexes suggested that the catalytic pocket consists of several important residues such as Ser_171_, Tyr_54_, Thr_101_, Ile_106_, Thr_107_, Phe_278_, His_281_, Gln_282_, Gln_289_, Ala_292_ and Phe_294,_ which are responsible for its catalytic activity and substrate binding. These residues fall on lid and flap domains and also consider the catalytic triad (Ser_171_, Asp_228_ and His_281_) interactions. The lid protects the active site and hence, is responsible for catalytic activity [15]. It has been predicted that the interaction with these catalytic pocket residues will significantly reduce the catalytic activity of Mrlip1. The selected compounds show close interactions with active site residue Ser_171_ and His_281_ of Mrlip1 (Figure 1, Figure 2 and Figure 3).

All these compounds are present in the deep cavity of Mrlip1 and mimick the binding pose with one another (Figure 1). The residues of Mrlip1 such as Ser_171_, Tyr_279_, Gln_282_, Arg_83_, and Thr_101_ form hydrogen bonds with the compounds in addition to several van der Waals and other interactions. Apart from these catalytic residues, many important interactions are offered by the catalytic pocket residues such as Tyr_54_, Asp_56_, Asn_77_, Thr_107_, Leu_172_, Phe_278_, His_281_, Gln_289_, Ala_292_, and Phe_294_ to the compounds to properly hold in the cavity of Mrlip1 (Figure 2 and Figure 3). Surface representations clearly indicated that the compounds reside in the internal part of the catalytic pocket and specifically bind with Mrlip1 important pocket residues with significant affinity (Figure 1B).

### 2.4. MD Simulation Data Analysis

To ensure the MD simulation data reproducibility and test the convergence of the result, the final production phase of each system was carried out with three independent MD set runs [16,17]. The replica has been shown as Run 1, Run 2, and Run 3 in Figures S1–S10. Furthermore, a detailed analysis was carried out using all trajectories.

#### 2.4.1. Average Potential Energy of Complex Systems

To ascertain the equilibration of the systems prior to MD analysis, the average potential energy of free Mrlip1, Mrlip1-RHC80267, Mrlip1-ZINC85530919, Mrlip1-ZINC95914464, and Mrlip1-ZINC85530320 were monitored. The constant temperature fluctuations at 300 K for each system suggest the stable and accurate nature of the MD simulations performed. The average potential energy for free Mrlip1, Mrlip1-RHC80267, Mrlip1-ZINC85530919, Mrlip1-ZINC95914464 and Mrlip1-ZINC85530320 were found to be −642,080 kJ/mol, −630,167 kJ/mol, −629,866 kJ/mol, −629,362 kJ/mol, and −632,500 kJ/mol, respectively.

#### 2.4.2. Conformational of Mrlip1

Binding of a compound in the catalytic pocket of the protein can lead to large conformational variations in a protein [18,19,20]. Root mean square deviation (RMSD) is one of the most important fundamental properties for establishing whether the protein is stable and close to the experimental structure [21]. The average RMSD values for free Mrlip1, Mrlip1-RHC80267, Mrlip1-ZINC85530919, Mrlip1-ZINC95914464 and Mrlip1-ZINC85530320 were found to be 0.25 nm, 0.21 nm, 0.27 nm, 0.20 nm, and 0.28 nm, respectively (Table 4).

**Figure 4:**
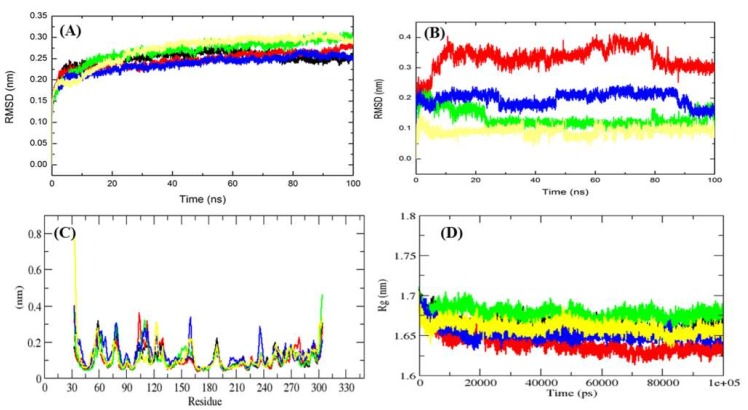
Dynamics of ligands binding to the Mrlip1. (**A**) RMSD plot for Mrlip1 as a function of time. (**B**) RMSD plot for ligands as a function of time. (**C**) Backbone residual fluctuations (RMSF) plot. (**D**) Time evolution of radius of gyration (*R*_g_). The values were presented during 100,000 ps (100 ns) MD simulations time scale. Black, red, green, blue, and yellow colors represent values obtained for Mrlip1, Mrlip1-RHC80267, Mrlip1-ZINC85530919, Mrlip1-ZINC95914464, and Mrlip1-ZINC85530320 complexes respectively.

The RMSD plot suggests that the binding of RHC80267 and ZINC95914464 stabilized the Mrlip1 and leads to less structural deviations from its native conformation (Figure 4A). In the case of the Mrlip1-ZINC85530919 and Mrlip1-ZINC85530320 complex, the binding of ZINC85530919 and ZINC85530320 to the Mrlip1 active pocket showed to have initial low fluctuations until 10–25 ns of the MD trajectories, thereafter it attained a high RMSD value. The orientation of ZINC85530919 and ZINC85530320 in the active pocket of Mrlip1 showed to have the least RMSD values and was found to be equilibrated during the 100 ns MD simulations. The RHC80267 and ZINC95914464 compounds showed continuous fluctuations in the active pocket of Mrlip1, which is possibly due to different orientations (Figure 4B).

**Figure 5:**
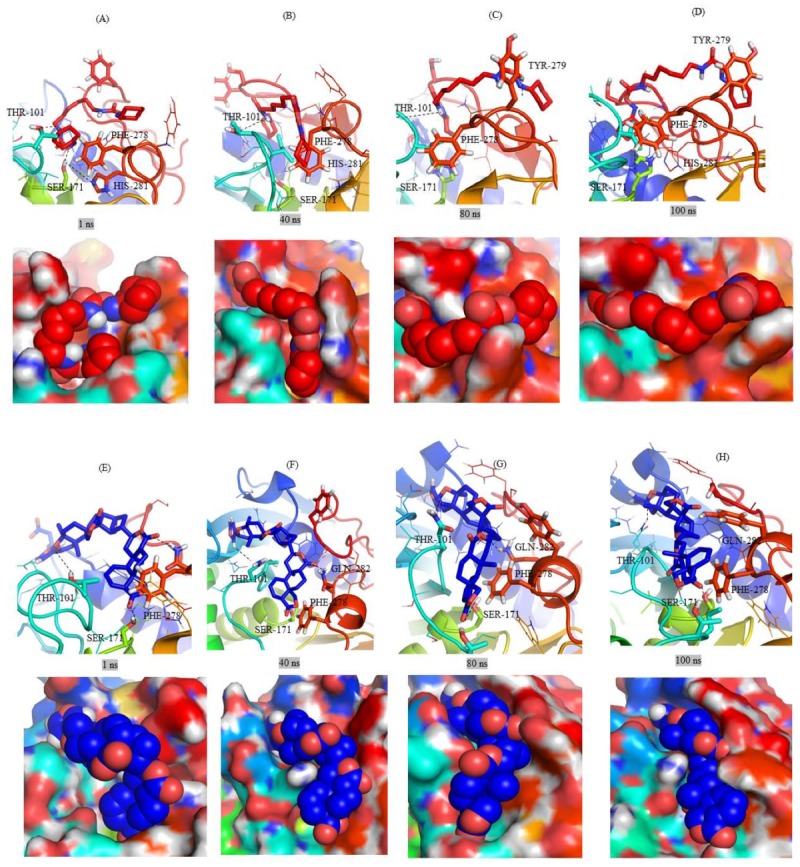

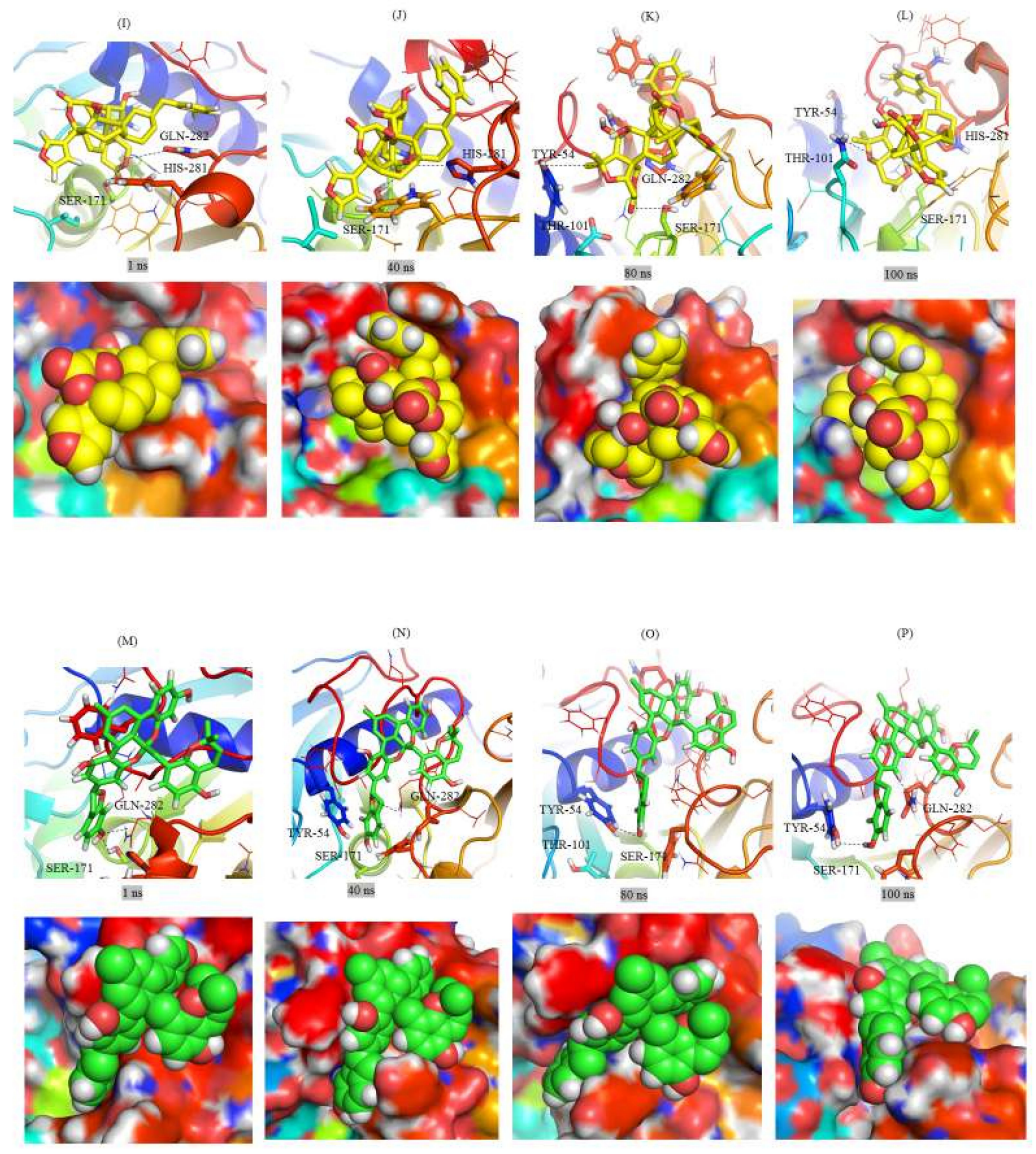
Ligands orientations during the different time scales. The MD trajectory of (**A**–**D**) Mrlip1-RHC80267, (**E**–**H**) Mrlip1-ZINC95914464, (I–L) Mrlip1-ZINC85530919, and (**M**–**P**) Mrlip1-ZINC85530320 complexes during different time intervals. The Mrlip1 is shown in cartoon as well as surface representation. Catalytic pocket residues are shown in the stick model.

Further analyses of each complex was carried out at different time intervals such as 1 ns, 40 ns, 80 ns and 100 ns. The orientations of RHC80267 in the catalytic pocket were totally different mainly due to the molecular structure of the compound. The known RHC80267 inhibitor contains two rings on both sides connected with flexible chains. Therefore, we observed one ring in the catalytic pocket and the other ring on the surface of the catalytic pocket (Figure 5A–D). On the other hand, ZINC95914464 compound also has a similar molecular structure, but it is not more flexible than RHC80267. These properties of the ZINC95914464 compound may make it a better drug-like molecule, since it occupied the catalytic pocket completely (Figure 5E–H). The ZINC85530919 and ZINC85530320 compounds showed similar orientation within the catalytic pocket. The catalytic pocket of Mrlip1 is highly selective in nature for the substrates. Therefore, very few interactions were formed by ZINC85530919 and ZINC85530320 compounds in the catalytic pocket (Figure 5I–L). Due to this reason, they cannot occupy the whole catalytic pocket. The replicas of all complexes showed a similar pattern of binding interaction with Mrlip1 (Figure S2).

Vibrations around the equilibrium are not random but depend on the local structure flexibility. To calculate the average fluctuation of all residues during the simulation, the root-mean square fluctuation (RMSF) of the Mrlip1 upon compounds binding was plotted as a function of residue number (Figure 4C). The RMSF plot showed that residual fluctuations are present in Mrlip1 in several regions of the protein structure. These residual fluctuations were found to be minimized upon binding of RHC80267 and ZINC95914464 and maximized with ZINC85530919 and ZINC85530320 at the region spanning the lid and flap domain.

Radius of gyration (*R_g_*) is a parameter linked to the tertiary structural volume of a protein and has been applied to obtain insight into the stability of a protein in a biological system. A protein is supposed to have a higher radius of gyration due to less tight packing. The average *R_g_* values for free Mrlip1, Mrlip1-RHC80267, Mrlip1-ZINC85530919, Mrlip1-ZINC95914464 and Mrlip1-ZINC85530320 were found to be 1.67 nm, 1.64 nm, 1.65 nm, 1.64 nm, and 1.67 nm, respectively. *R_g_* plot suggested that the Mrlip1 attained more tight packing in Mrlip1-RHC80267 and Mrlip1-ZINC95914464, and when bound to Native, ZINC85530919 and then ZINC85530320 (Figure 4D).

#### 2.4.3. Solvent Accessible Surface Area

**Figure 6:**
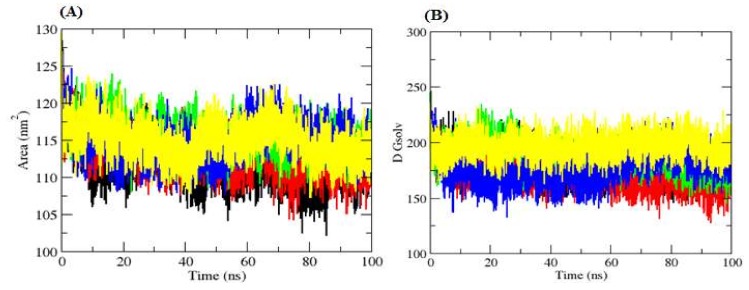
Solvent Accessible Surface Area. (**A**) The SASA plot. (**B**) The free energy of solvation. The Black, red, green, blue, and yellow color represent values obtained for Mrlip1, Mrlip1-RHC80267, Mrlip1-ZINC85530919, Mrlip1-ZINC95914464, and Mrlip1-ZINC85530320 respectively.

Solvent Accessible Surface Area (SASA) is defined as the surface area of a protein which interacts with its solvent molecules [22]. Average SASA values for free Mrlip1, Mrlip1-RHC80267, Mrlip1-ZINC85530919, Mrlip1-ZINC95914464 and Mrlip1-ZINC85530320 complexes were monitored during 100 ns MD simulations (Figure 6). The average SASA values for free Mrlip1, Mrlip1-RHC80267, Mrlip1-ZINC85530919, Mrlip1-ZINC95914464 and Mrlip1-ZINC85530320 complexes were found to be 133.16 nm^2^, 134.71 nm^2^, 133.69 nm^2^, 134.76 nm^2^, and 132.77 nm^2^, respectively. There was no major change observed in the SASA values due to ligands binding. During SASA calculations, the free energy of solvation of free Mrlip1, Mrlip1-RHC80267, Mrlip1-ZINC85530919, Mrlip1-ZINC95914464, and Mrlip1-ZINC85530320 was calculated. The free energy of solvation of free Mrlip1, Mrlip1-RHC80267, Mrlip1-ZINC85530919, Mrlip1-ZINC95914464, and Mrlip1-ZINC85530320 was found to be 181.83 kJ/mol/nm^2^, 172.17 kJ/mol/nm^2^, 187.16 kJ/mol/nm^2^, 177.10 kJ/mol/nm^2^, and 196.83 kJ/mol/nm^2^, respectively. The free energy of solvation was found to be high for Mrlip1-ZINC85530320; while Mrlip1, Mrlip1-RHC80267, Mrlip1-ZINC85530919 and Mrlip1-ZINC95914464 showed lower values.

#### 2.4.4. Hydrogen Bonds Analysis

**Figure 7:**
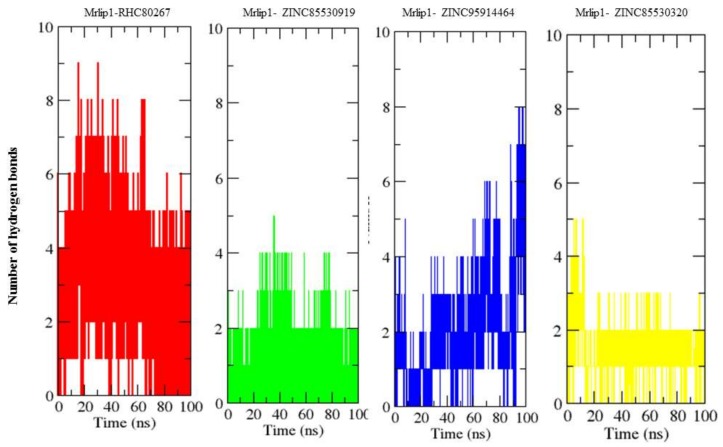
Hydrogen bonds analysis. The average number of hydrogen bonds between Mrlip1-RHC80267, Mrlip1-ZINC85530919, Mrlip1-ZINC95914464, and Mrlip1-ZINC85530320 as a function of time. The values were presented during 100,000 ps (100 ns) MD simulations time scale. The red, green, blue, and yellow color represent values obtained for Mrlip1-RHC80267, Mrlip1-ZINC85530919, Mrlip1-ZINC95914464, and Mrlip1-ZINC85530320 complexes respectively.

Hydrogen bonding between a protein and compounds provides a directionality and specificity of interaction that is an important aspect for molecular recognition [23]. In order to validate the stability of the docked complexes, the hydrogen bonds paired within 0.35 nm between Mrlip1 and compounds in Mrlip1-RHC80267, Mrlip1-ZINC85530919, Mrlip1-ZINC95914464 and Mrlip1-ZINC85530320 were calculated in a solvent environment during the MD simulations. We found that RHC80267 and ZINC95914464 strongly bound to the active pocket of Mrlip1 with 2–6 hydrogen bonds, while ZINC85530919 and ZINC85530320 bound to the active pocket of Mrlip1 with 1–3 hydrogen bonds with least fluctuations (Figure 7).

#### 2.4.5. Secondary Structure Changes upon Ligands Binding

**Figure 8:**
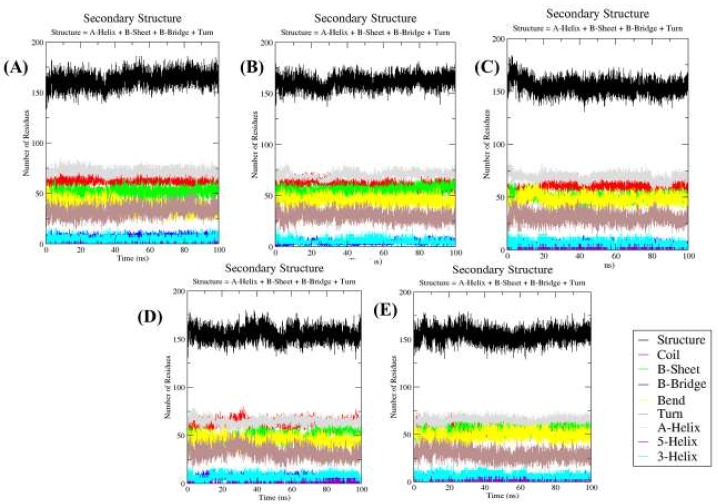
Secondary structure plot. The secondary structure analysis indicating the structural elements present in (**A**) Mrlip1, (**B**) Mrlip1-RHC80267, (**C**) Mrlip1-ZINC85530919, (**D**) Mrlip1-ZINC95914464, and (**E**) Mrlip1-ZINC85530320 during the 100 ns MD simulations.

The purpose of this analysis is to measure the secondary structure content of a protein as a function of time. The secondary structure assignments in protein such as the α-helix, β-sheet and turn were fragmented into individual residues for each time step. The average number of residues that participated in secondary structure formation in the case of complexes was slightly decreased due to an increase in the percentage of coils and a decrease in the β-sheet in comparison with Mrlip1 (Table 5). There was a slight decrease in the percentage of the β-sheet, and an α-helix was found in the case of Mrlip1-ZINC85530919 (Figure 8).

### 2.5. Principal Component Analysis

**Figure 9:**
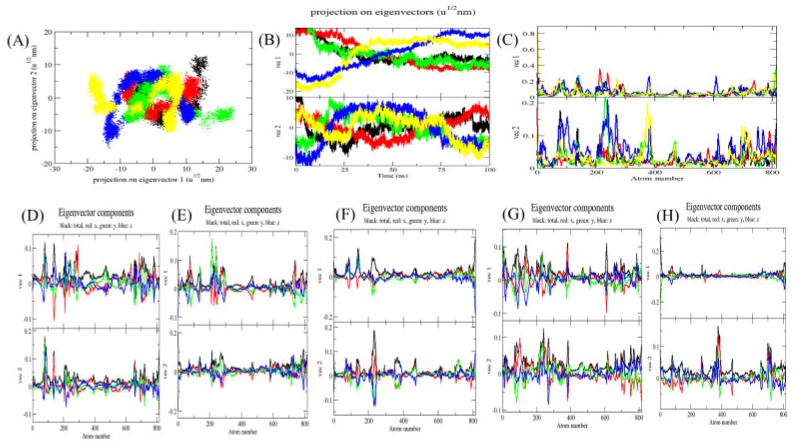
Principal Component Analysis. (**A**) The 2D projections of trajectories on eigenvectors showed different projections of Mrlip1. (**B**) The projections of trajectories on eigenvectors with respect to time. (**C**) Root mean square atomic fluctuations (RMSF) obtained during PCA calculations. Black, red, green, blue and yellow colour represent values obtained for Mrlip1, Mrlip1-RHC80267, Mrlip1-ZINC85530919, Mrlip1-ZINC95914464 and Mrlip1-ZINC85530320 respectively. The Eigenvector components were further resolved for Mrlip1 into total (black), x (red), y (green), and z (blue) directions for (**D**) Mrlip1, (**E**) Mrlip1-RHC80267, (**F**) Mrlip1-ZINC85530919, (**G**) Mrlip1-ZINC95914464, and (**H**) Mrlip1-ZINC85530320, respectively.

The principal component analysis (PCA) or essential dynamics (ED) reflects the overall expansion of a protein during different simulation [24]. In this method, the dynamics of Mrlip1 was calculated using *gmx covar* module with respect to the backbone. PCA identifies the large scale average motion of a protein, thus revealing the structures underlying the atomic fluctuations [25]. The sum of the eigenvalues is a measure of the total motility in the system. It can be used to compare the flexibility of a protein under different conditions [26]. The trace of the covariance matrix and eigenvalues for free Mrlip1, Mrlip1-RHC80267, Mrlip1-ZINC85530919, Mrlip1-ZINC95914464, and Mrlip1-ZINC85530320 were found to be 131.214 nm^2^, 146.472 nm^2^, 151.465 nm^2^, 212.819 nm^2^, and 196.618 nm^2^, respectively. The eigenvalues for free Mrlip1 were found to be low in comparison with complexes that clearly indicate that a random fluctuation increases upon compounds binding; and Mrlip1-ZINC95914464 showed higher eigenvalues, which indicates a higher expansion of Mrlip1 i.e., low compactness of complexes at 300 *K*. The multidimensional atomic covariance matrix for each atom pair covariance is depicted (Figure 9). The gmx anaeig module reads a set of eigenvectors and eigenvalues as input files and returns to project an MD trajectory along selected eigenvector values. The 2D projections of trajectories on eigenvectors showed overlap between free Mrlip1, Mrlip1-RHC80267, and Mrlip1-ZINC95914464 complexes. The results also suggested that the difference in the position of atoms is due to the binding of complexes with Mrlip1. The root mean square atomic fluctuations of Mrlip1 were also recorded during the PCA calculations. The Eigenvector components were further resolved into x, y and z directions.

### 2.6. Gibbs Free Energy Landscape

**Figure 10:**
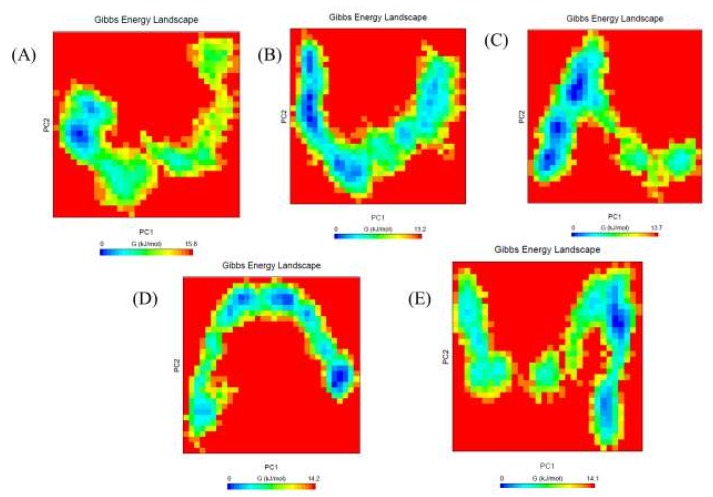
The Gibbs free energy landscape. The Gibbs free energy landscape plot obtained during 100 ns MD simulations for (**A**) Mrlip1, (**B**) Mrlip1-RHC80267, (**C**) Mrlip1-ZINC85530919, (**D**) Mrlip1-ZINC95914464, and **(E)** Mrlip1-ZINC85530320 respectively.

The Gibbs free energy landscape was also calculated using *gmx covar*, *gmx anaeig*, and *gmx sham* using the projections of their own first (PC1) and second (PC2) eigenvectors, respectively. The color-coded Gibbs free energy landscape is also depicted in Figure 10. Gibbs free energy landscape inspects the direction of the fluctuation in the two systems for all Cα atoms of the free Mrlip1, Mrlip1-RHC80267, Mrlip1-ZINC85530919, Mrlip1-ZINC95914464, and Mrlip1-ZINC85530320 complex structures from trajectories. The corresponding free energy contour map with a deeper blue color indicates a lower energy. It has been found that the main free energy well in the global free energy minimum region was completely changed after binding of these compounds. These free energies well indicate the stable conformational states of this molecule. The binding of these compounds to Mrlip1 leads to different global minima of Mrlip1 during the 100 ns MD simulations. The energy landscape exhibits several clearly distinguishable minima, which correspond to the metastable conformational states that are separated with a small energy barrier. The most metastable conformational states were seen in the binding of Mrlip1-RHC80267, Mrlip1-ZINC85530919, and Mrlip1-ZINC95914464, in which local minima distributed to about three to four regions within the energy landscape. The Free Mrlip1 and Mrlip1-ZINC85530320 formed just two to three metastable conformations during the whole population of trajectories (Figure S10).

### 2.7. MMPBSA Binding Energy Analysis

The extraction of binding energies from the stable short region of 40 to 50 ns simulation, 200 trajectory frames were used for the *mmpbsa* calculations using polar and apolar solvation parameters. The aim of this analysis is to establish the energies associated with the binding of compounds to Mrlip1 during the MD simulations. The average binding energy for Mrlip1-RHC80267, Mrlip1-ZINC85530919, Mrlip1-ZINC95914464 and Mrlip1-ZINC85530320 were found to be −98.51 kJ/mol, −232.28 kJ/mol, −183.17 kJ/mol, and −85.71 kJ/mol, respectively (Table 6).

The average binding energy was found to be stronger in the case of Mrlip1-ZINC95914464. This may be due to the stable orientation of ZINC95914464 in the active pocket of Mrlip1 as deduced from the RMSD plot. The average binding energy in the case of control inhibitor Mrlip1-RHC80267 was comparatively less than the potential screened compound ZINC95914464.

## 3. Materials and Methods

This work was carried out on an HPC server of Intel^®^ Xeon^®^ CPU ES-2673 v4 @ 2.30 GHz (Intel Corporation, Guangzhou, China), which has 40 logical cores and 30 TB data space. Several computational tools were used in this study. The AutoDock Vina [27] and PyRx 0.8 (FastSpring, Amesterdam, Netherlands) were used for vHTS and molecular docking studies. PyMOL [28], VMD (visual molecular dynamics) [29] and Discovery studio visualizer [30] were used for visualization purposes. In addition, the online web-servers and data repository resources like NCBI, PDB, ZINC database, PubChem, SwissADME [31], and CarcinoPred-EL [32] were used for retrieval, evaluation and analysis of the data.

### 3.1. Preparation of Target Protein and Natural Compounds Library

The three-dimensional (3D) structure of open conformation of Mrlip1 was obtained from our previous work [33]. The open conformation of Mrlip1 was validated by well-studied *Rhizomucor miehei* lipase (PDB ID: 4TGL) which was co-crystalized with DEP (Di-Ethyl-Phosphonate) in its catalytic pocket. The same pose and orientation of DEP was modelled in Mrlip1 lipase and used for vHTS. A library of natural compounds based on the TCM database containing 60,000 small compounds was obtained from non-commercial ZINC database [34]. The library contains processed chemical structures of TCM natural compounds in 3D file formats which were used in this study to perform structure-based vHTS.

### 3.2. Hit Selection and Drug-Ability Evaluation

Based on binding affinities and scoring, the top 80 hits were selected, which showed higher binding-affinity towards Mrlip1. The selected compounds were further analyzed based on physicochemical and ADME properties to find out safe and effective drug-like compounds. These properties including toxicity and carcinogenicity were predicted using web-based applications such as Swiss-ADME [31], PreADMET and CarcinoPred-EL [32]. Further, the selected compounds were checked for a PAINS (Pan-assay interference compounds) pattern using the SwissADME web server, (accessed on: 23 June 2018) [35] and ZINC^15^ chemistry pattern database, (accessed on: 25 June 2018) [36] and then further interaction examinations were carried out to avoid a false positive and get selective compounds with high specificity towards the binding pocket of Mrlip1.

### 3.3. Mrlip1 Structural Analysis: Visualization and Evaluation

The visual inspection of docked conformations of compounds with Mrlip1 was performed using PyMOL and Discovery Studio visualizer LigPlot^+^ visualization tools. These softwares create high quality animated 3D as well as 2D figures of Mrlip1 and chemical compounds. Apart from the visual inspections, numerous measurements such as bond length, distance between residues, and distance between the Mrlip1 and compounds were calculated.

### 3.4. MD Simulations

Molecular dynamics (MD) simulation is a convenient method to understand the chemistry of biological macromolecules at the molecular level [37,38]. Many mysterious biological functions in proteins and their profound dynamic mechanisms can be revealed by studying their internal motions [39,40,41,42]. MD simulations were performed on free Mrlip1, Mrlip1-RHC80267, Mrlip1-ZINC85530919, Mrlip1-ZINC95914464, and Mrlip1-ZINC85530320 at 300 K at the molecular mechanics level implemented in the GROMACS 5.1.2 [43] using the GROMOS96 43a1 force-field [44]. The compounds topology and force-field parameters were generated using the PRODRG server [45]. Charges in the topology file were manually corrected. The additional compound atoms were merged in the complex topology files, and the parameters for all the compounds were included in the system topology.

The free Mrlip1, Mrlip1-RHC80267, Mrlip1-ZINC85530919, Mrlip1-ZINC95914464, and Mrlip1-ZINC85530320 were soaked in a cubic box of water molecules with an initial dimension of 8 nm, i.e., setting the box edge 1 nm from the molecule periphery using the gmx editconf module for creating boundary conditions and *gmx solvate* module for solvation. The Simple Point Charge (spc216) water model was used to solvate the protein. The charges on the free Mrlip1 and its complexes were neutralized by the addition of Na^+^ and Cl^−^ ions using the *gmx genion* module to maintain neutrality, preserving a physiological concentration (0.15 M). Energygrps in the molecular dynamics parameters (mdp) file was used to examine the interactions between Mrlip1 with compounds and the particle-mesh Ewald method [46] was applied. Then, the MD system was minimized by means of steepest descent (1500 steps). The temperature was then raised (0 to 300 K) during the equilibration period of 100 ps under periodic boundary conditions at a constant volume.

Equilibration was completed in two constant stages such as NVT (number of particles, volume, and temperature) ensemble and NPT (number of particles, pressure, and temperature at 100 ps) ensemble. The final production phase (100 ns) was achieved thereafter at 300 *K*. Several Gromacs modules were used to analyze the MD trajectories such as gmx energy, gmx rms, gmx confirms, gmx rmsf, gmx gyrate, make_ndx, gmx hbond, gmx anaeig, gmx sham, gmx do_dssp, gmx_covar, and gmx sasa. The graphical presentation of the 3D models was prepared using VMD (Visual Molecular Dynamics) [29] and PyMOL.

### 3.5. MMPBSA Calculation

Molecular mechanics Poisson Boltzmann surface area (MMPBSA) is an attractive approach which has been used effectively to reproduce and justify experimental findings and to improve the results of virtual screening and docking [47]. A short MD trajectory was extracted from the stable region of each complex for MMPBSA estimations [48]. The binding energy was calculated using MMPBSA approaches of the g_mmpbsa package [49]. It was calculated using the following equation:

ΔG_Binding_ = G_Complex_ − (G_Protein_ + G_Ligand_)

where, G_Complex_ signifies the total free energy of the binding complex, and G_Protein_ and G_Ligand_ are the measures of the total free energies of the individual protein and compounds, respectively.

## 4. Conclusions

Identifying potential and specific inhibitors for Mrlip1 is a promising approach to address skin diseases. In this study, the Traditional Chinese Medicine database has been screened against *Malassezia restricta* lipase (Mrlip1) to find promising and highly effective anti-dandruff inhibitors. The possible inhibitors of Mrlip1 such as ZINC85530919, ZINC95914464 and ZINC85530320 were selected on the basis of high binding affinities, interactions with catalytic triad residues, and predicted activity scoring values. Based on several screenings, three best hits were identified and are expected to be possible inhibitors of Mrlip1. Various examinations such as drug-likeliness, ADME and toxicity were applied on all three compounds. All selected compounds interact with catalytic pocket residues and occupy the same binding pocket as RHC80267, a well-known diacylglycerol lipase inhibitor. These compounds have a better average binding affinity. The MD simulation study revealed that these compounds formed stable conformations within the catalytic pocket and exhibit different conformational changes in the Mrlip1. Amongst all the identified inhibitors, ZINC95914464 was found the best stability in the catalytic pocket. Therefore, ZINC95914464 can be a new inhibitor that could serve as the starting point for the development of more promising anti-dandruff therapeutic agents.

## Figures and Tables

**Table 1:** Physicochemical properties of the selected compounds *.

S. No.	Compound ID	MW	LogP	HBD	HBA	rBonds
1.	RHC80267^©^	394.51	5.42	2	8	13
2.	ZINC85627948	721.99	4.18	7	8	2
3.	ZINC85530320	682.8	4.20	1	9	3
4.	ZINC85569100	676.82	4.89	7	7	9
5.	ZINC95914464	670.79	4.35	0	9	2
6.	ZINC95914661	690.92	4.64	4	8	3
7.	ZINC85545357	597.87	3.96	3	7	1
8.	ZINC95914660	691.93	5.03	5	7	3
9.	ZINC85542923	552.85	4.90	4	3	0
10.	ZINC85632555	701.87	2.56	5	9	4
11.	ZINC85545857	662.94	4.22	5	6	2
12.	ZINC85546208	618.8	4.53	3	5	3
13.	ZINC85569586	710.94	3.77	3	7	1
14.	ZINC85632546	715.9	2.37	5	9	4
15.	ZINC85569094	720.91	5.48	7	7	8
16.	ZINC85531411	688.8	4.09	1	8	4
17.	ZINC85569130	674.84	5.25	6	6	8
18.	ZINC85546197	670.88	4.39	3	5	3
19.	ZINC85570604	684.99	5.00	4	5	2
20.	ZINC85530916	644.71	3.98	4	8	3
21.	ZINC85530919	644.71	4.05	4	8	3
22.	ZINC85542736	691.02	6.17	4	3	2
23.	ZINC85570874	723.98	4.74	4	7	7
24.	ZINC85570863	750.02	4.76	4	7	7
25.	ZINC85542639	715.04	6.46	4	3	3

* MW. Molecular weight (Dalton), LogP: Lipophilicity, HBD: Hydrogen bond donor, HBA: Hydrogen bond acceptor, and rBonds: Rotatable bonds (measure of molecular flexibility of a compound). © symbol denoted as control inhibitor compound.

**Table 2:** ADMET properties of the selected compounds *.

S. No.	Molecule	BBB Perme Ant	PPB	HIA	TPSA	Log S	Skin Permea Bility	CYP2D6 Inhibitor	Carcinogenicity
1.	RHC80267^©^	No	88.69	89.79	101.38	−5.27	−3.40	No	NC
2.	ZINC85530916	No	100.00	94.70	117.84	−9.52	−2.49	No	NC
3.	ZINC85530919	No	100.00	94.70	117.84	−9.52	−2.49	No	NC
4.	ZINC85531411	No	92.38	97.27	112.27	−9.96	−1.69	No	NC
5.	ZINC85546197	No	97.26	96.06	86.99	−9.30	−1.02	No	NC
6.	ZINC85546208	No	95.88	95.90	86.99	−8.30	−1.48	No	NC
7.	ZINC85545357	No	90.33	94.84	129.39	−7.94	−3.14	No	NC
8.	ZINC85545857	No	97.47	90.39	118.22	−9.05	−2.51	No	NC
9.	ZINC85530320	No	91.92	98.24	124.8	−7.32	−2.00	No	NC
10.	ZINC95914464	No	89.91	99.27	110.5	−5.52	−4.20	No	NC
11.	ZINC95914660	No	69.28	94.49	62.28	−8.69	−3.36	No	NC
12.	ZINC85569586	No	95.00	95.81	105.45	−6.59	−1.10	No	NC
13.	ZINC85570604	No	100.00	93.72	97.99	−7.96	−3.27	No	NC
14	ZINC85570874	No	96.46	94.37	133.38	−9.87	−1.03	No	NC

* BBB (Blood brain barrier) penetration ability, PPB (Plasma protein binding): >85% is strongly bound, HIA (Human intestinal absorption): 70–100% is for well absorbed compounds, PSA (Polar surface area): ≤90 Å^2^ is the optimum value, LogS: water solubility, and NC (Non-carcinogen). © symbol denoted as control inhibitor compound. ADMET values predicted using SwissADME (http://www.swissadme.ch/, accessed on: 23 June 2018) and PreADMET (https://preadmet.bmdrc.kr/, accessed on: 25 June 2018). Carcinogenicity was predicted using CarcinoPred-EL (http://ccsipb.lnu.edu.cn/toxicity/CarcinoPred-EL/, accessed on: 25 June 2018).

**Table 3:** List of selected compounds with their interacting residues of Mrlip1.

S. No	Compound IDs	Catalytic Pocket Interacting Residues		
		Hydrogen Bonds	π-π	π-Alkyl
1.	RHC80267^©^	Ser_101_, Asn_102_, Gln_282_	-	Tyr_54_, Ile_106_, Phe_278_, Tyr_279_
2.	ZINC85530919	Ser_171_, Tyr_279_, Gln_282_	Phe_278_, Phe_294_, Tyr_279_	Phe_278_, Tyr_279_
3.	ZINC85530320	Ser_171_, Gln_282_	Phe_294_	Tyr_54_, Phe_278_
4.	ZINC95914464	Ser_171_, Arg_83_, Thr_101_, His_281_	-	Ala_292_, Tyr_54_, Phe_278_

**©** symbol denoted as control inhibitor compound.

**Table 4:** The calculated parameters for all the system obtained after 100 ns MD simulations.

Complexes	Average Potential Energy (kJ/mol)	Radius of Gyration (nm)	Average RMSD (nm)	Average SASA (Backbone, nm^2^)	Free Energy of Solvation (kJ/mol/nm^2^)
Mrlip1	−642,080	1.67	0.25	132.15	181.83
Mrlip1-RHC80267^©^	−630,167	1.64	0.21	134.81	172.17
Mrlip1-ZINC85530919	−629,866	1.67	0.27	133.69	187.16
Mrlip1-ZINC95914464	−629,362	1.65	0.20	134.06	177.10
Mrlip1-ZINC85530320	−632,500	1.67	0.28	132.77	196.83

**©** Symbol denoted as control inhibitor compound.

**Table 5:** Percentage of residues that participated in average structure formation.

Complexes	Percentage of Protein Secondary Structure (SS%)							
	Structure *	Coil	β-sheet	β-bridge	Bend	Turn	α-helix	3_10_-helix
Mrlip1	60	23	19	2	14	12	26	2
Mrlip1-RHC80267^©^	59	22	21	2	17	11	26	2
Mrlip1-ZINC85530919	57	22	18	2	18	11	25	3
Mrlip1-ZINC95914464	57	23	19	2	16	12	24	3
Mrlip1-ZINC85530320	57	21	21	1	18	11	24	3

* Structure = α-helix + β-sheet + β-bridge + Turn, © symbol denoted as control inhibitor compound.

**Table 6:** Calculated MMPBSA binding energy between Mrlip1 lipase and their potential inhibitors.

S. No.	Complexes	Average Binding Energy (kJ/mol)
1.	Mrlip1-RHC80267^©^	−98.51 ± 12.97
2.	Mrlip1-ZINC85530919	−232.28 ± 16.27
3.	Mrlip1-ZINC95914464	−183.17 ± 10.98
4.	Mrlip1-ZINC85530320	−85.71 ± 12.53

**©** Symbol denoted as control inhibitor compound.

## Supplementary Materials

Supplementary materials can be found at http://www.mdpi.com/1422-0067/20/4/884/s1.

## Abbreviations

DAG	Diacyl glyceride
MMPBSA	Molecular mechanics Poisson Boltzmann surface area
TCM	Traditional Chinese Medicine
vTHS	Virtual High-throughput Screening

## References

[1] Cao M., Fonseca L.M., Schoenfuss T.C., Rankin S.A. (2014). Homogenization and lipase treatment of milk and resulting methyl ketone generation in blue cheese. J. Agric. Food Chem..

[2] Nomura D.K., Long J.Z., Niessen S., Hoover H.S., Ng S.W., Cravatt B.F. (2010). Monoacylglycerol lipase regulates a fatty acid network that promotes cancer pathogenesis. Cell.

[3] Xu D., Sun L., Chen H., Lan D., Wang Y., Yang B. (2012). Enzymatic synthesis of diacylglycerols enriched with conjugated linoleic acid by a novel lipase from Malassezia globosa. J. Am. Oil Chem. Soc..

[4] Khan F.I., Nizami B., Anwer R., Gu K.R., Bisetty K., Hassan M.I., Wei D.Q. (2017). Structure prediction and functional analyses of a thermostable lipase obtained from Shewanella putrefaciens. J. Biomol. Struct. Dyn..

[5] Sommer B., Overy D., Kerr R.G. (2015). Identification and characterization of lipases from Malassezia restricta, a causative agent of dandruff. FEMS Yeast Res..

[6] Park M., Jung W.H., Han S.H., Lee Y.H., Lee Y.W. (2015). Characterisation and Expression Analysis of MrLip1, a Class 3 Family Lipase of Malassezia restricta. Mycoses.

[7] Xu Z., Wang Z., Yuan C., Liu X., Yang F., Wang T., Wang J., Manabe K., Qin O., Wang X. (2016). Dandruff is associated with the conjoined interactions between host and microorganisms. Sci. Rep..

[8] Gemmer C.M., DeAngelis Y.M., Theelen B., Boekhout T., Dawson T.L. (2002). Fast, noninvasive method for molecular detection and differentiation of Malassezia yeast species on human skin and application of the method to dandruff microbiology. J. Clin. Microbiol..

[9] DeAngelis Y.M., Gemmer C.M., Kaczvinsky J.R., Kenneally D.C., Schwartz J.R., Dawson T.L. (2005). Three etiologic facets of dandruff and seborrheic dermatitis: Malassezia fungi, sebaceous lipids, and individual sensitivity. J. Investig. Dermatol. Symp. Proc..

[10] Schofield D.A., Westwater C., Warner T., Balish E. (2005). Differential Candida albicans lipase gene expression during alimentary tract colonization and infection. FEMS Microbiol. Lett..

[11] Stehr F., Felk A., Gácser A., Kretschmar M., Mähnss B., Neuber K., Hube B., Schäfer W. (2004). Expression analysis of the Candida albicans lipase gene family during experimental infections and in patient samples. FEMS Yeast Res..

[12] Brunke S., Hube B. (2006). MfLIP1, a gene encoding an extracellular lipase of the lipid-dependent fungus Malassezia furfur. Microbiology.

[13] Soares R.C., Zani M.B., Arruda A.C., Arruda L.H., Paulino L.C. (2015). Malassezia intra-specific diversity and potentially new species in the skin microbiota from Brazilian healthy subjects and seborrheic dermatitis patients. PLoS ONE.

[14] Veber D.F., Johnson S.R., Cheng H.Y., Smith B.R., Ward K.W., Kopple K.D. (2002). Molecular properties that influence the oral bioavailability of drug candidates. J. Med. Chem..

[15] Khan F.I., Lan D., Durrani R., Huan W., Zhao Z., Wang Y. (2017). The Lid Domain in Lipases: Structural and Functional Determinant of Enzymatic Properties. Front. Bioeng. Biotechnol..

[16] Monhemi H., Housaindokht M.R., Moosavi-Movahedi A.A., Bozorgmehr M.R. (2014). How a protein can remain stable in a solvent with high content of urea: Insights from molecular dynamics simulation of Candida antarctica lipase B in urea: Choline chloride deep eutectic solvent. Phys. Chem. Chem. Phys..

[17] Knapp B., Ospina L., Deane C.M. (2018). Avoiding False Positive Conclusions in Molecular Simulation: The Importance of Replicas. J. Chem. Theory Comput..

[18] Gramany V., Khan F.I., Govender A., Bisetty K., Singh S., Permaul K. (2016). Cloning, expression, and molecular dynamics simulations of a xylosidase obtained from Thermomyces lanuginosus. J. Biomol. Struct. Dyn..

[19] Khan F.I., Shahbaaz M., Bisetty K., Waheed A., Sly W.S., Ahmad F., Hassan M.I. (2016). Large scale analysis of the mutational landscape in beta-glucuronidase: A major player of mucopolysaccharidosis type VII. Gene.

[20] Khan F.I., Wei D.Q., Gu K.R., Hassan M.I., Tabrez S. (2016). Current updates on computer aided protein modeling and designing. Int. J. Biol. Macromol..

[21] Kuzmanic A., Zagrovic B. (2010). Determination of ensemble-average pairwise root mean-square deviation from experimental B-factors. Biophys. J..

[22] Mazola Y., Guirola O., Palomares S., Chinea G., Menéndez C., Hernández L., Musacchio A. (2015). A comparative molecular dynamics study of thermophilic and mesophilic beta-fructosidase enzymes. J. Mol. Model..

[23] Hubbard R.E., Kamran Haider M. (2001). Hydrogen Bonds in Proteins: Role and Strength.

[24] Maisuradze G.G., Liwo A., Scheraga H.A. (2009). Principal component analysis for protein folding dynamics. J. Mol. Biol..

[25] David C.C., Jacobs D.J. (2014). Principal component analysis: A method for determining the essential dynamics of proteins. Methods Mol. Biol..

[26] Tiana G., Simona F., De Mori G.M., Broglia R.A., Colombo G. (2004). Understanding the determinants of stability and folding of small globular proteins from their energetics. Protein Sci..

[27] Trott O., Olson A.J. (2010). AutoDock Vina: Improving the speed and accuracy of docking with a new scoring function, efficient optimization, and multithreading. J. Comput. Chem..

[28] Warren D. (2002). The PyMOL Molecular Graphics System. http://pymol.sourceforge.net/overview/index.htm.

[29] Humphrey W., Dalke A., Schulten K. (1996). VMD: Visual molecular dynamics. J. Mol. Graph..

[30] Biovia D.S. (2015). Discovery Studio Modeling Environment.

[31] Daina A., Michielin O., Zoete V. (2017). SwissADME: A free web tool to evaluate pharmacokinetics, drug-likeness and medicinal chemistry friendliness of small molecules. Sci. Rep..

[32] Zhang L., Ai H., Chen W., Yin Z., Hu H., Zhu J., Zhao J., Zhao Q., Liu H. (2017). CarcinoPred-EL: Novel models for predicting the carcinogenicity of chemicals using molecular fingerprints and ensemble learning methods. Sci. Rep..

[33] Ali S., Khan F.I., Chen W., Rahaman A., Wang Y. (2018). Open and closed states of Mrlip1 DAG lipase revealed by molecular dynamics simulation. Mol. Simul..

[34] Irwin J.J., Shoichet B.K. (2005). ZINC—A free database of commercially available compounds for virtual screening. J. Chem. Inf. Model..

[35] Baell J.B., Holloway G.A. (2010). New substructure filters for removal of pan assay interference compounds (PAINS) from screening libraries and for their exclusion in bioassays. J. Med. Chem..

[36] Sterling T., Irwin J.J. (2015). ZINC 15–ligand discovery for everyone. J. Chem. Inf. Model..

[37] Khan S., Khan F.I., Mohammad T., Khan P., Hasan G.M., Lobb K.A., Islam A., Ahmad F., Hassan M.I. (2018). Exploring molecular insights into the interaction mechanism of cholesterol derivatives with the Mce4A: A combined spectroscopic and molecular dynamic simulation studies. Int. J. Biol. Macromol..

[38] Syed S.B., Khan F.I., Khan S.H., Srivastava S., Hasan G.M., Lobb K.A., Islam A., Ahmad F., Hassan M.I. (2018). Mechanistic insights into the urea-induced denaturation of kinase domain of human integrin linked kinase. Int. J. Biol. Macromol..

[39] Khan F.I., Aamir M., Wei D.Q., Ahmad F., Hassan M.I. (2017). Molecular mechanism of Ras-related protein Rab-5A and effect of mutations in the catalytically active phosphate-binding loop. J. Biomol. Struct. Dyn..

[40] Anwer K., Sonani R., Madamwar D., Singh P., Khan F., Bisetty K., Ahmad F., Hassan M.I. (2015). Role of N-terminal residues on folding and stability of C-phycoerythrin: Simulation and urea-induced denaturation studies. J. Biomol. Struct. Dyn..

[41] Stephens D.E., Khan F.I., Singh P., Bisetty K., Singh S., Permaul K. (2014). Creation of thermostable and alkaline stable xylanase variants by DNA shuffling. J. Biotechnol..

[42] Wang Y.J., Khan F.I., Xu Q., Wei D.Q. (2018). Recent Studies of Mitochondrial SLC25, Integration of Experimental and Computational Approaches. Curr. Protein Pept. Sci..

[43] Van Der Spoel D., Lindahl E., Hess B., Groenhof G., Mark A.E., Berendsen H.J. (2005). GROMACS: Fast, flexible, and free. J. Comput. Chem..

[44] Pol-Fachin L., Fernandes C.L., Verli H. (2009). GROMOS96 43a1 performance on the characterization of glycoprotein conformational ensembles through molecular dynamics simulations. Carbohydr. Res..

[45] Schuttelkopf A.W., van Aalten D.M. (2004). PRODRG: A tool for high-throughput crystallography of protein-ligand complexes. Acta Crystallogr. D Biol. Crystallogr..

[46] Norberto de Souza O., Ornstein R.L. (1999). Molecular dynamics simulations of a protein-protein dimer: Particle-mesh Ewald electrostatic model yields far superior results to standard cutoff model. J. Biomol. Struct. Dyn..

[47] Genheden S., Ryde U. (2015). The MM/PBSA and MM/GBSA methods to estimate ligand-binding affinities. Expert Opin. Drug Discov..

[48] Homeyer N., Gohlke H. (2012). Free Energy Calculations by the Molecular Mechanics Poisson-Boltzmann Surface Area Method. Mol. Inform..

[49] Kumari R., Kumar R., Lynn A. (2014). g_mmpbsa—A GROMACS Tool for High-Throughput MM-PBSA Calculations. J. Chem. Inf. Model..

