# Supplementary material for: Identification and Evaluation of Inhibitors of Lipase from Malassezia restricta using Virtual High-Throughput Screening and Molecular Dynamics Studies
Source: Int J Mol Sci. 2019 Feb 18;20(4):884. doi: 10.3390/ijms20040884 (PMC6412828; doi:10.3390/ijms20040884)
Supplement: Supplementary file 1 [file ijms-20-00884-s001.zip › supplementary_Figures.pdf]

Supplementary Data

Identification and evaluation of inhibitors of lipase from *Malassezia restricta* using virtual High-throughput screening and molecular dynamics studies

Shahid Ali[1], Faez Iqbal Khan[2], Taj Mohammad[3], Dongming Lan[1], Md. Imtaiyaz Hassan[3] and Yonghua Wang[1*]

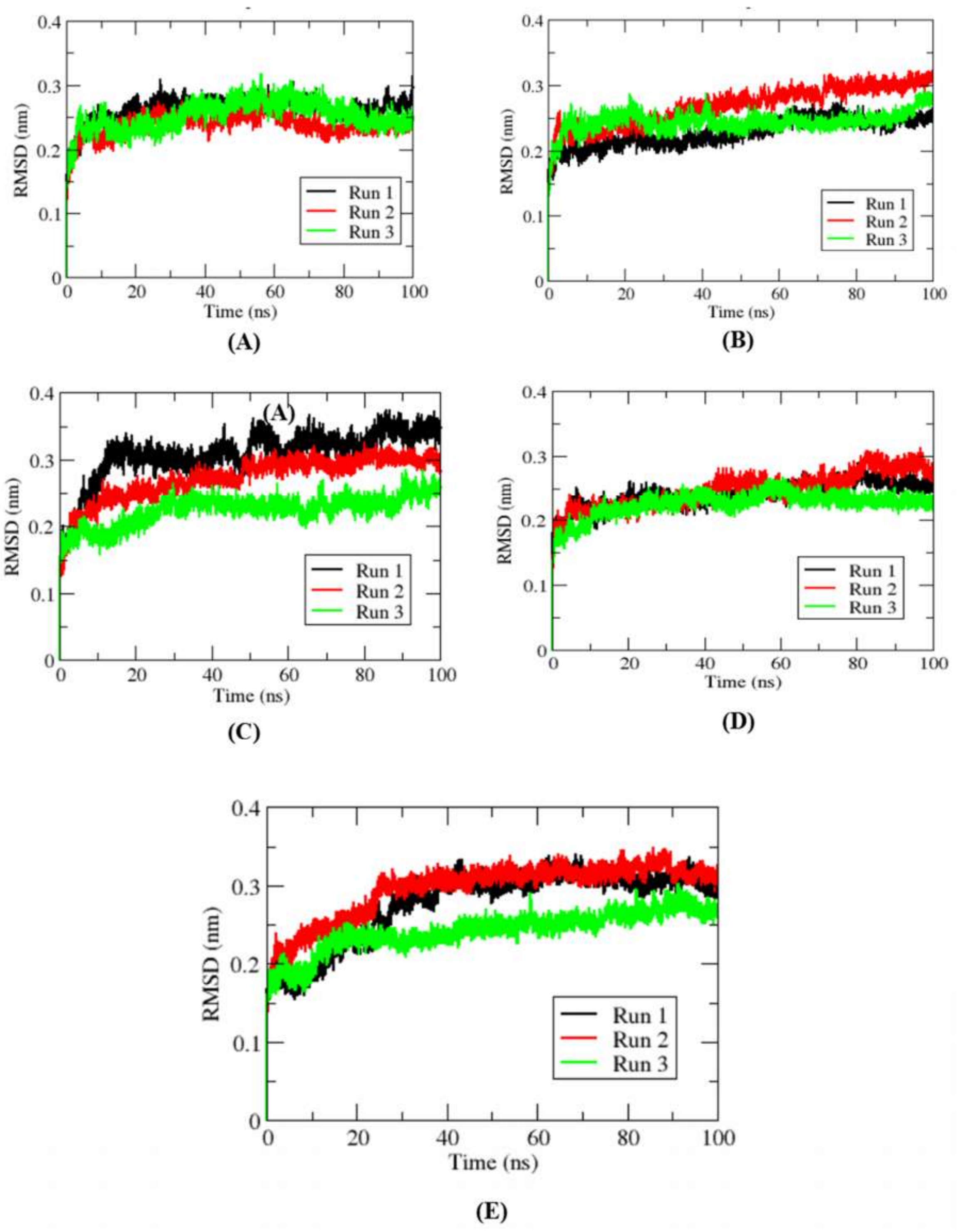

Figure S1. Convergence of each sets of MD results of protein. RMSD plot of Mrlip1(Backbone) for each sets of MD simulation as a function of time. (A) free Mrlip1, (B) Mrlip1-RHC80267, (C) Mrlip1-ZINC85530919, (D) Mrlip1- ZINC95914464 and (E) Mrlip1- ZINC85530320. The values were presented during 100,000 ps (100 ns) MD simulations time scale.

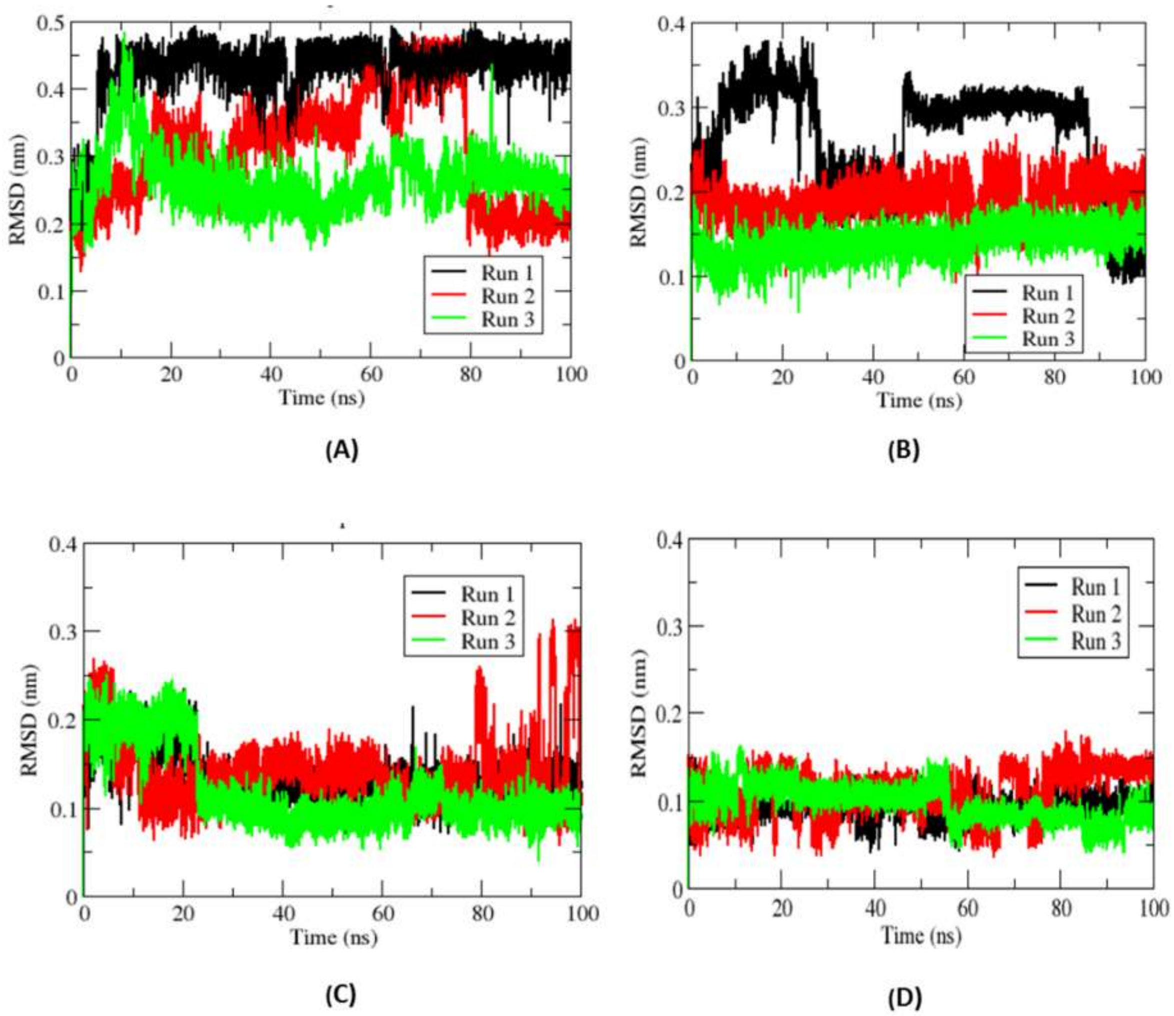

Figure S2. Convergence of each sets of MD results of ligands. RMSD plot of ligands within the catalytic pocket of Mrlip1 as a function of time. (A) RHC80267, (B) ZINC95914464 (C) ZINC85530919, and (D) ZINC85530320. The values were presented during 100,000 ps (100 ns) MD simulations time scale.

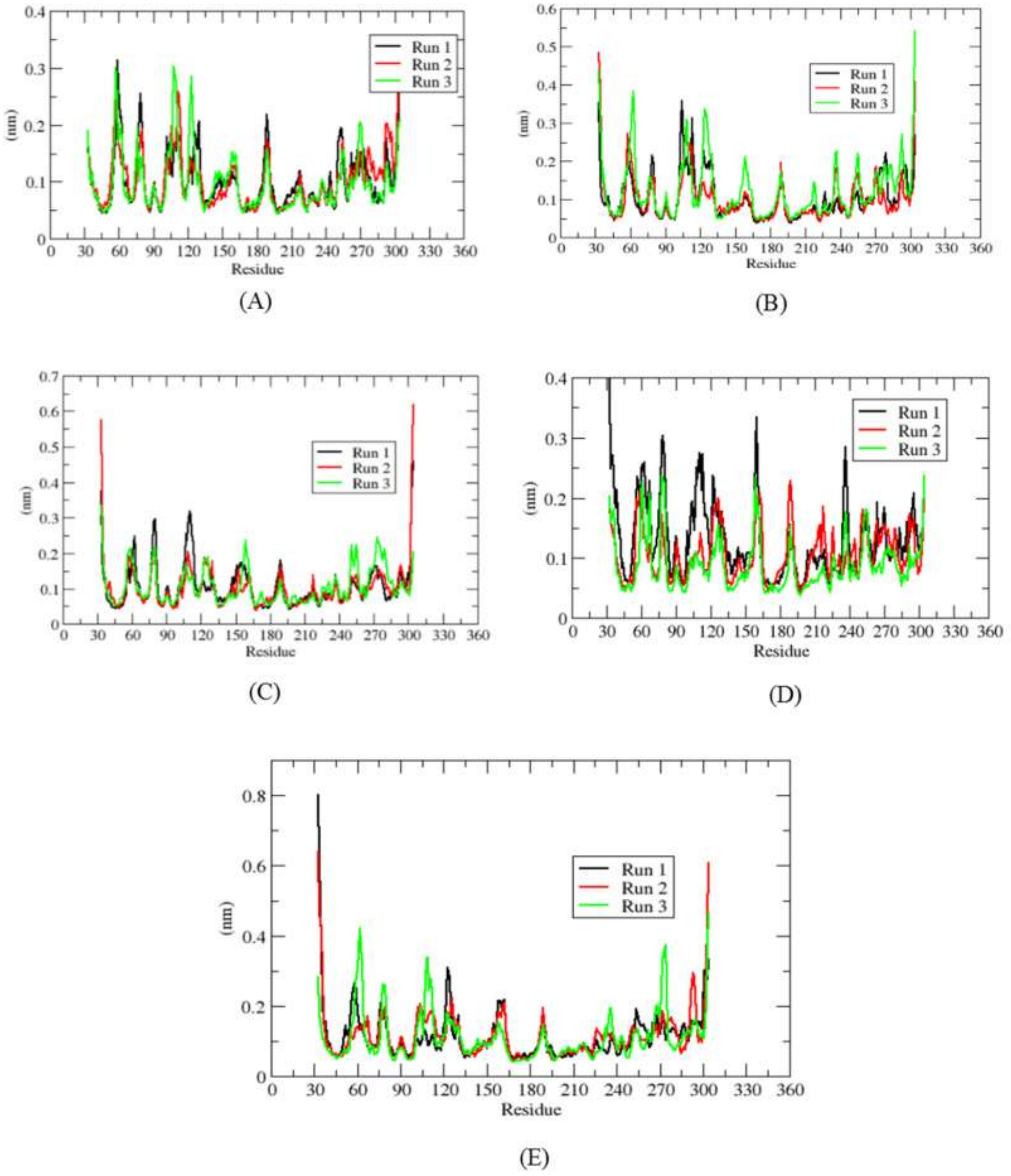

Figure S3. Convergence of each sets of MD results of protein. RMSF plot of Mrlip1(Backbone) for each sets of MD simulation as a function of time. (A) free Mrlip1, (B) Mrlip1-RHC80267, (C) Mrlip1-ZINC85530919, (D) Mrlip1- ZINC95914464 and (E) Mrlip1- ZINC85530320. The values were presented during 100,000 ps (100 ns) MD simulations time scale.

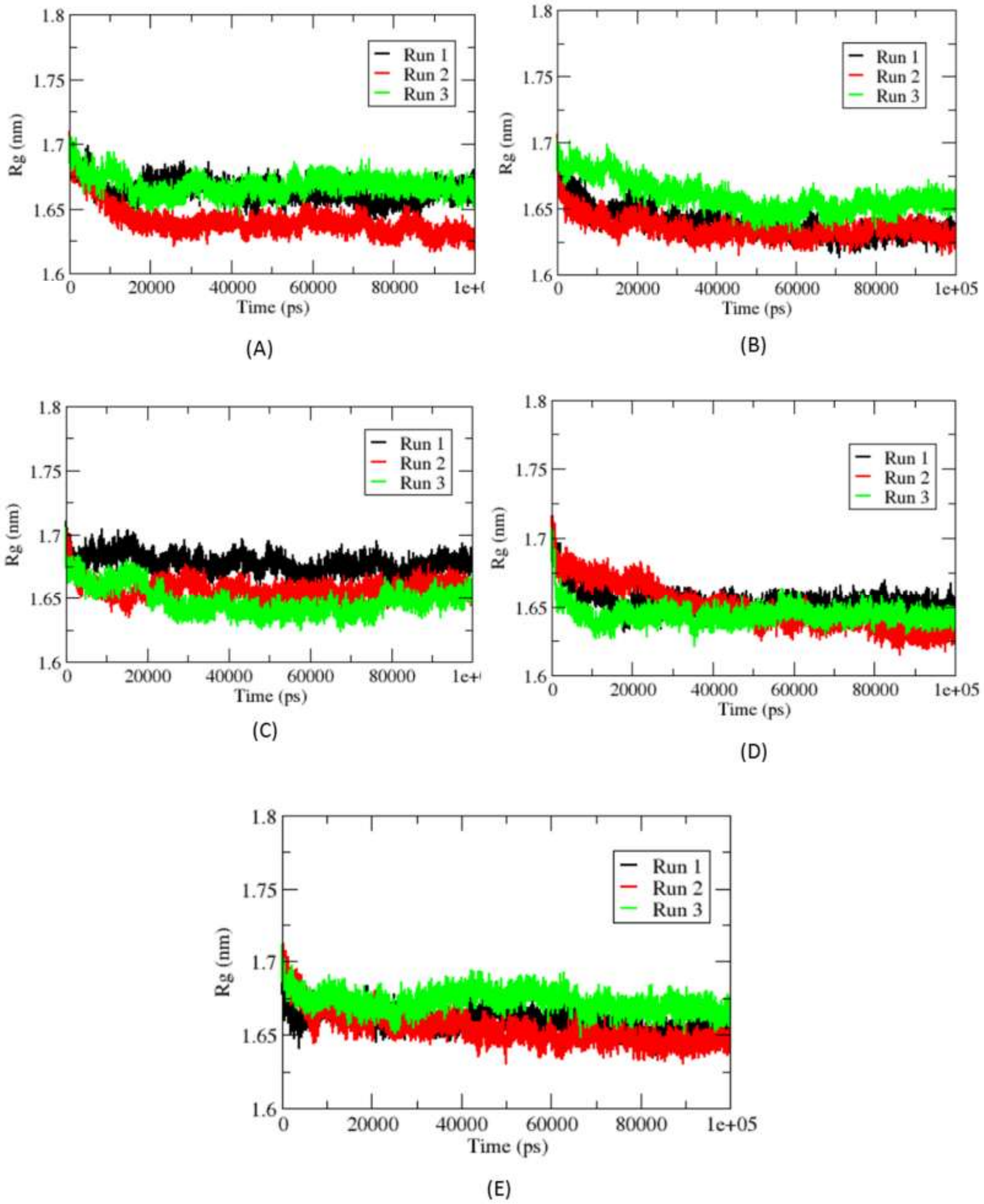

Figure S4. Convergence of each sets of MD results of protein. Radius of gyration plot of Mrlip1 for each sets of MD simulation as a function of time. (A) free Mrlip1, (B) Mrlip1-RHC80267, (C) Mrlip1-ZINC85530919, (D) Mrlip1- ZINC95914464 and (E) Mrlip1- ZINC85530320. The values were presented during 100,000 ps (100 ns) MD simulations time scale.

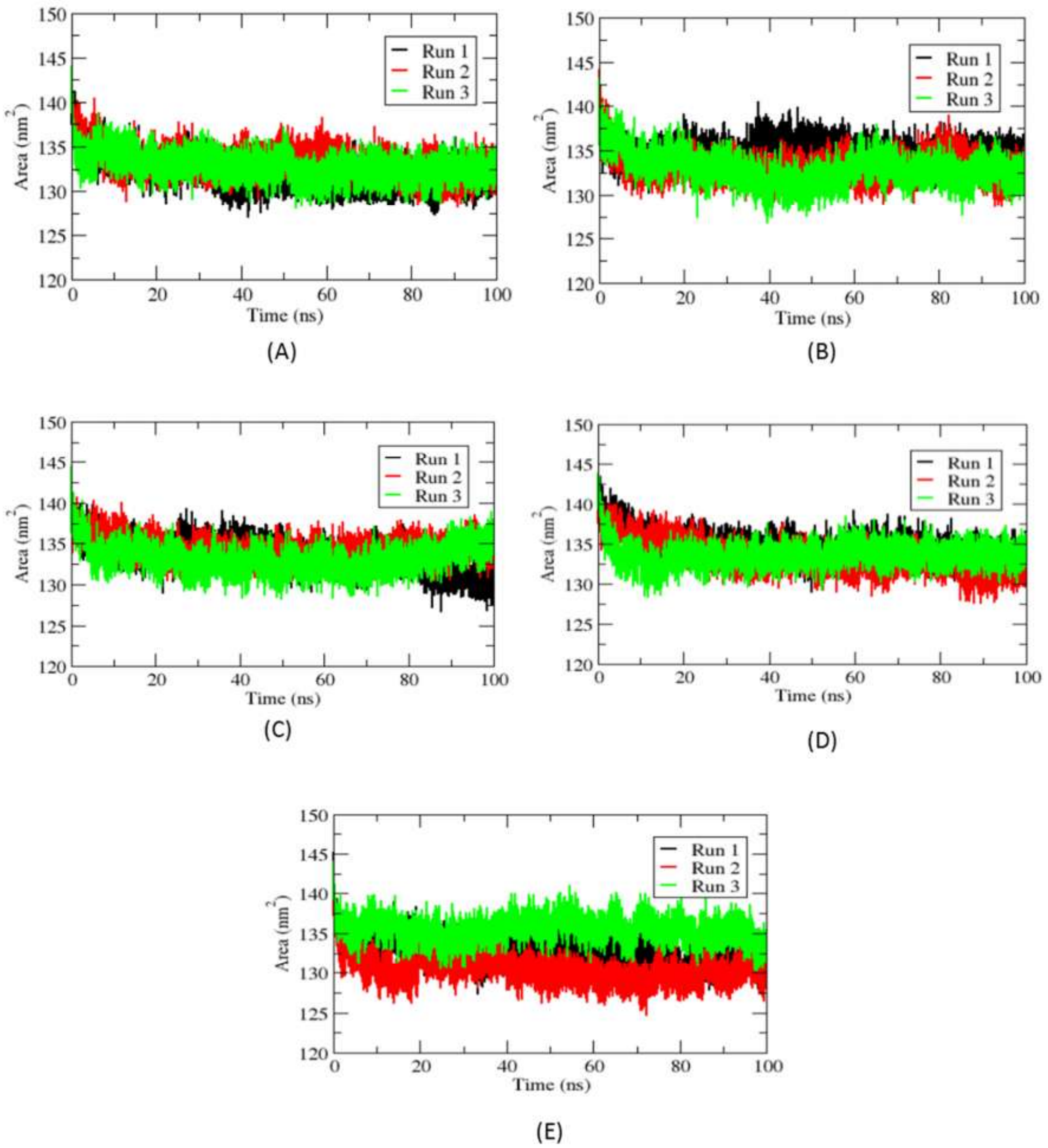

Figure S5. Convergence of each sets of MD results of protein. SASA plot of Mrlip1 for each sets of MD simulation as a function of time. (A) free Mrlip1, (B) Mrlip1-RHC80267, (C) Mrlip1- ZINC85530919, (D) Mrlip1- ZINC95914464 and (E) Mrlip1- ZINC85530320. The values were presented during 100,000 ps (100 ns) MD simulations time scale.

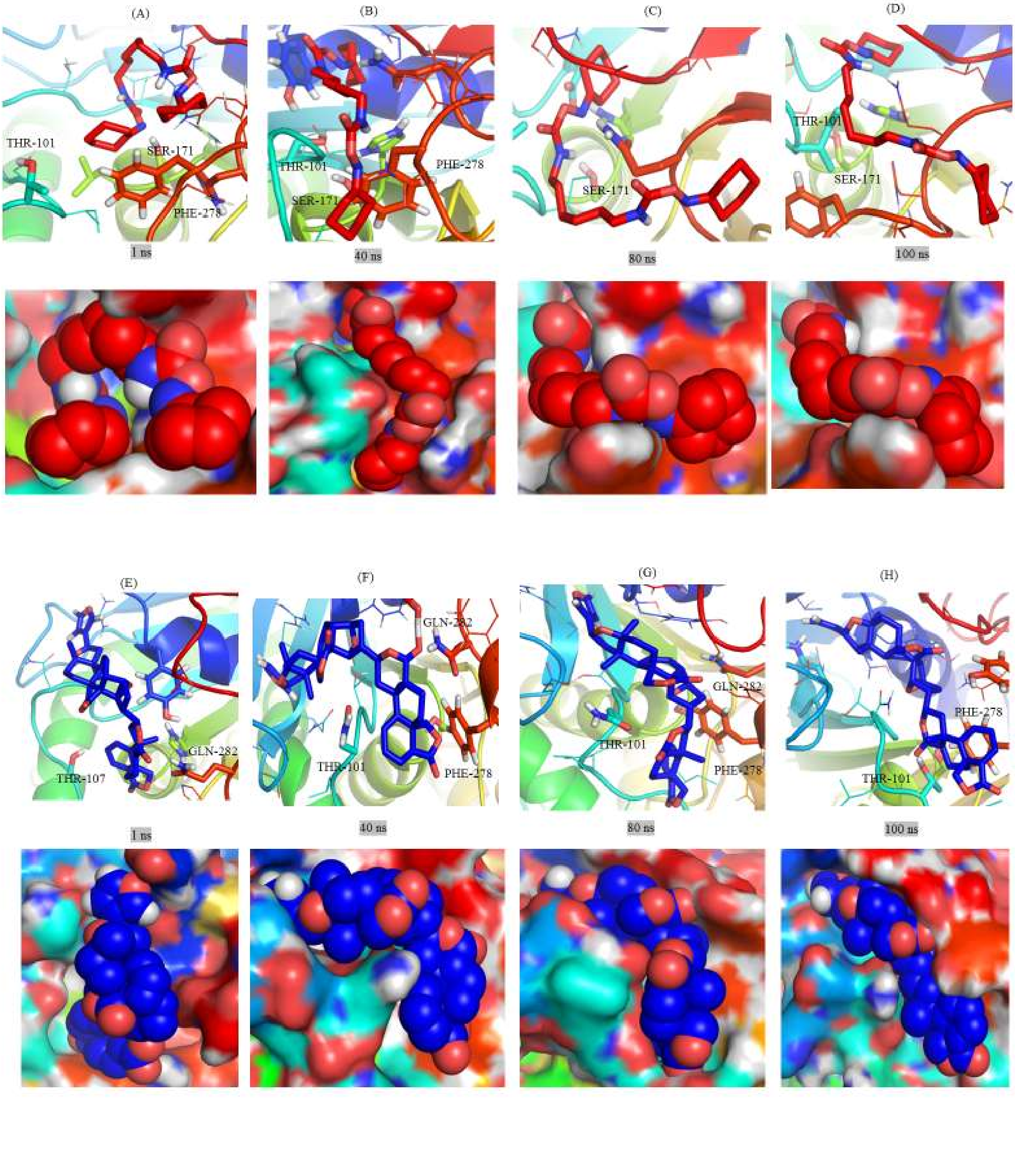
(A)
(B)
(C)
(D)
THR-101
SER-171
PHE-278
1 ns
THR-101
SER-171
PHE-278
40 ns
SER-171
80 ns
THR-101
SER-171
100 ns
(E)
(F)
(G)
(H)
GLN-282
THR-107
1 ns
GLN-282
THR-101
PHE-278
40 ns
GLN-282
THR-101
PHE-278
80 ns
PHE-278
THR-101
100 ns

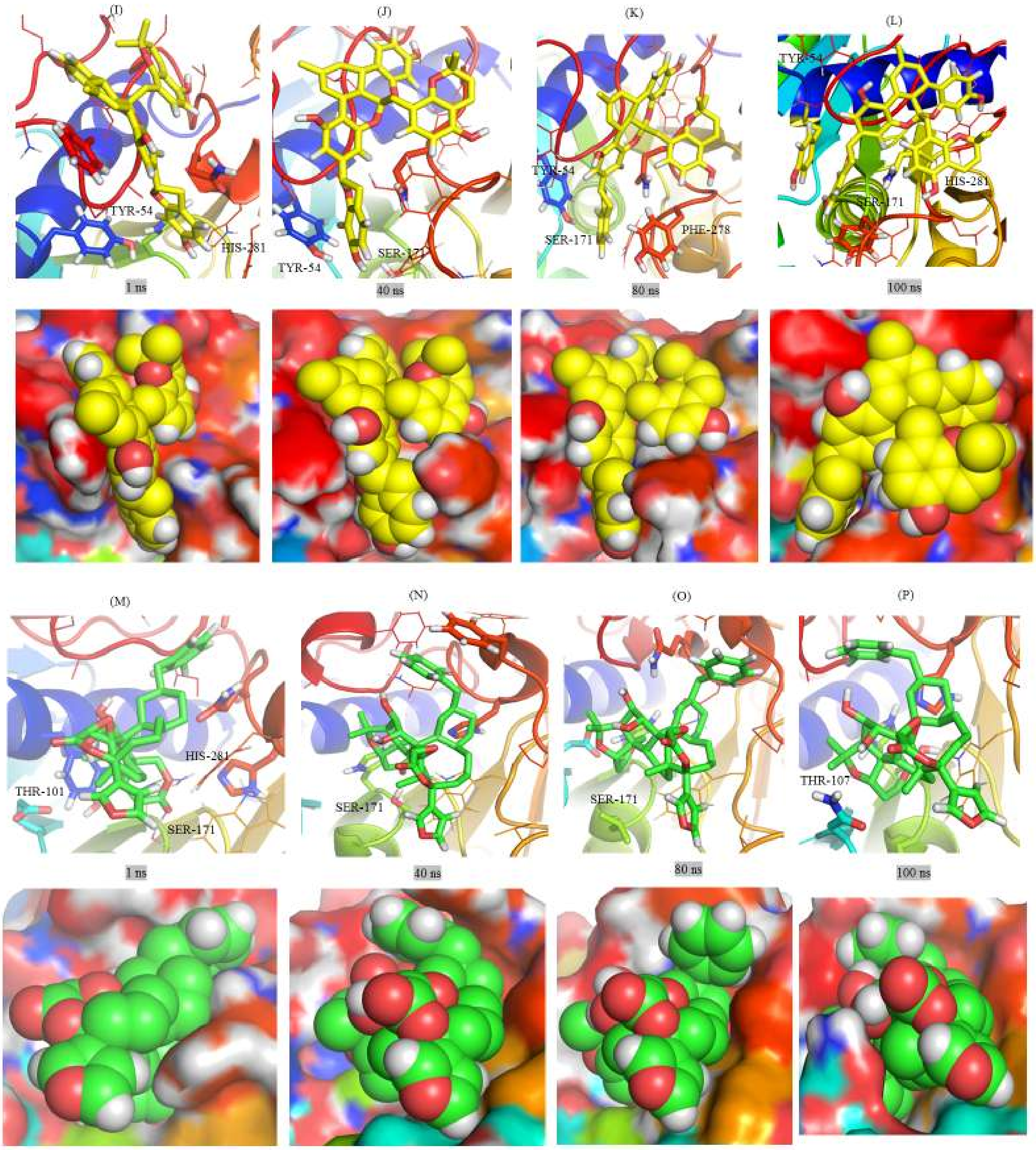

Figure S6. Ligands orientations during the different time scales (Replica). The MD trajectory of (A-D) Mrlip1- RHC80267, (E-H) Mrlip1- ZINC95914464, (I-L) Mrlip1- ZINC85530919, and (M-P) Mrlip1- ZINC85530320 complex during different time intervals. The Mrlip1 shown in cartoon as well as surface representation. Catalytic pocket residues were shown in stick model.

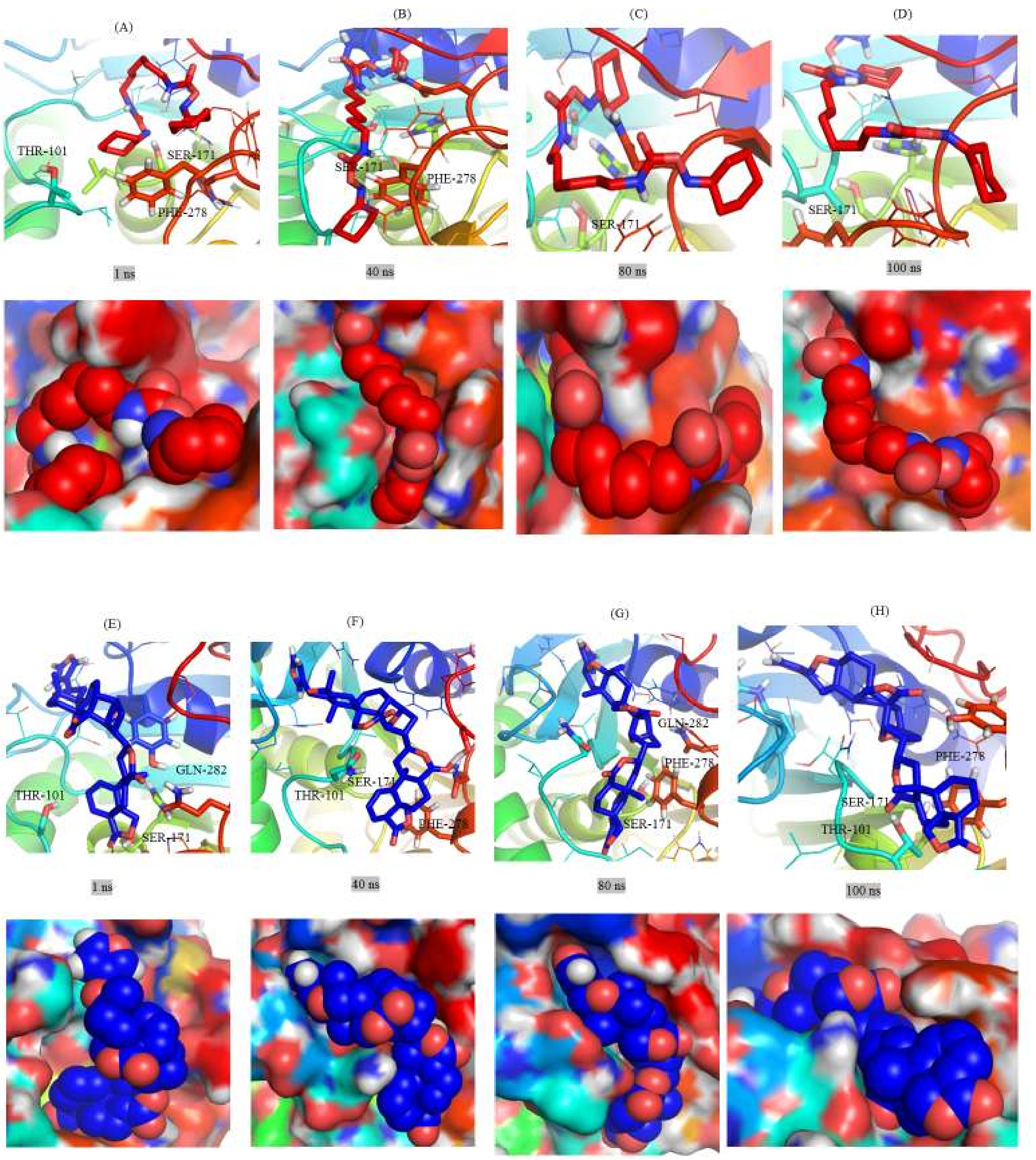

(A)
(B)
(C)
(D)
THR-101
SER-171
PHE-278
SER-171
PHE-278
SER-171
SER-171
1 ns
40 ns
80 ns
100 ns
(E)
(F)
(G)
(H)
GLN-282
THR-101
SER-171
SER-171
THR-101
PHE-278
GLN-282
PHE-278
SER-171
PHE-278
SER-171
THR-101
1 ns
40 ns
80 ns
100 ns

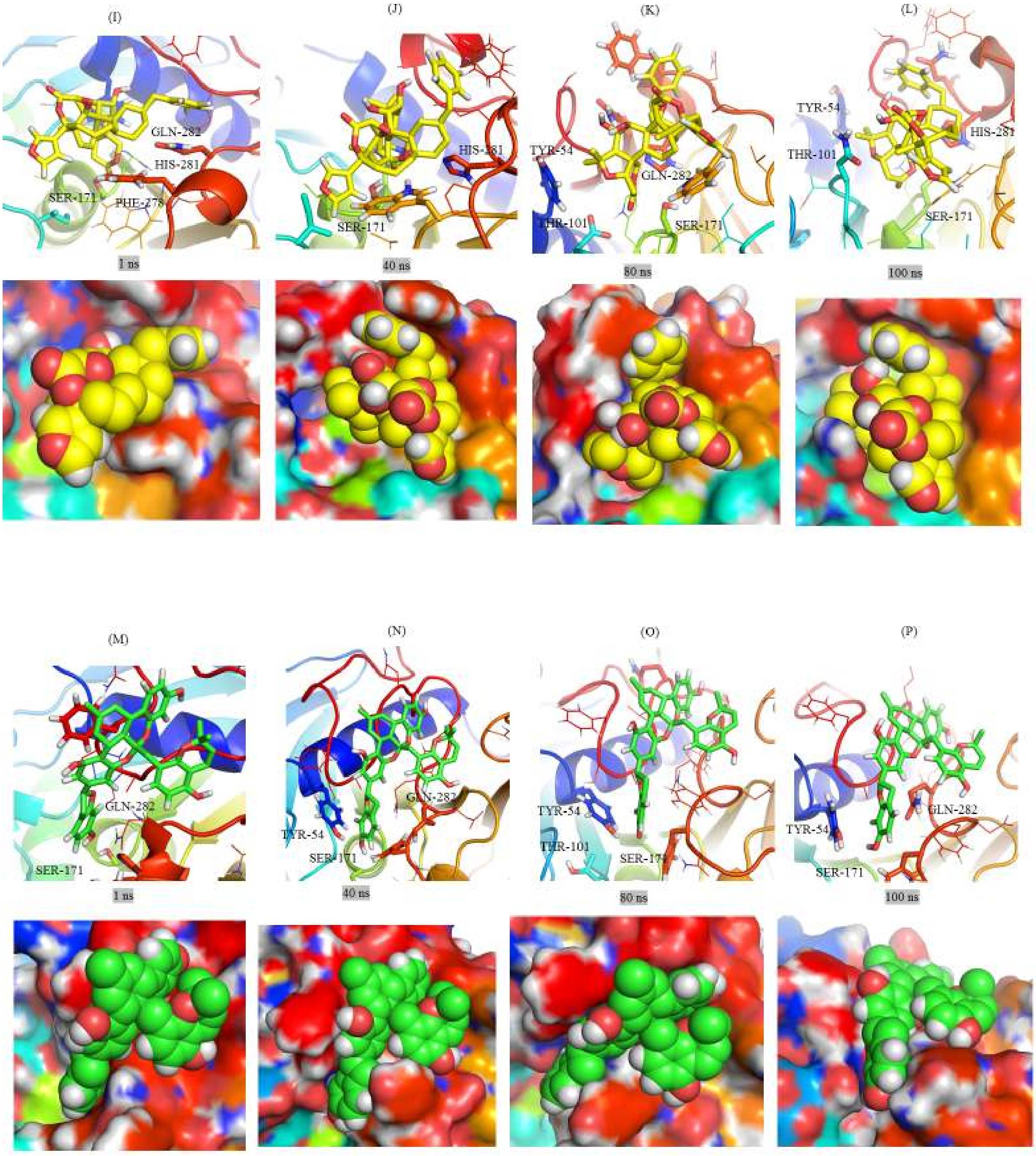

Figure S7. Ligands orientations during the different time scales (Replica). The MD trajectory of (A-D) Mrlip1-RHC80267, (E-H) Mrlip1- ZINC95914464, (I-L) Mrlip1- ZINC85530919, and (M-P) Mrlip1- ZINC85530320 complex during different time intervals. The Mrlip1 shown in cartoon as well as surface representation. Catalytic pocket residues were shown in stick model.

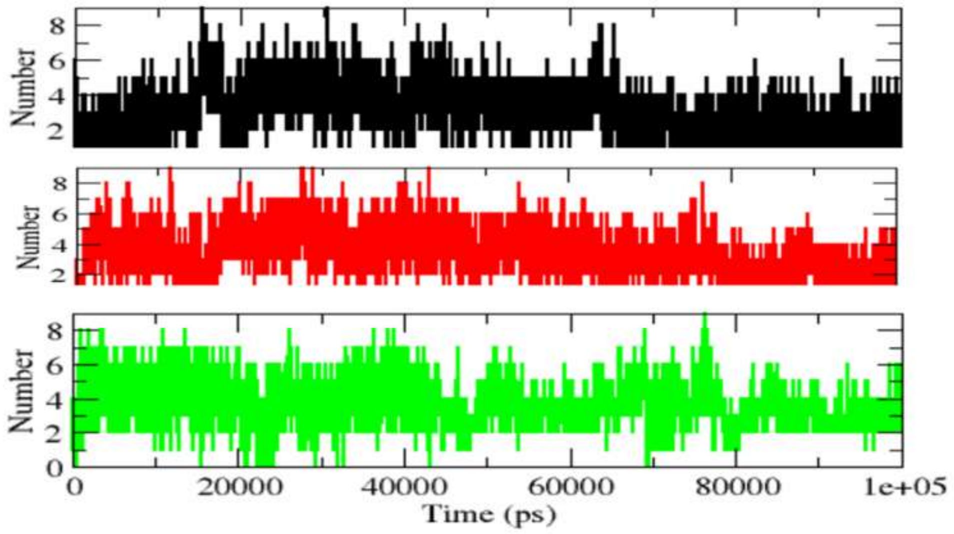

Number
Number
Number
Time (ps)
0
20000
40000
60000
80000
1e+05

(A)

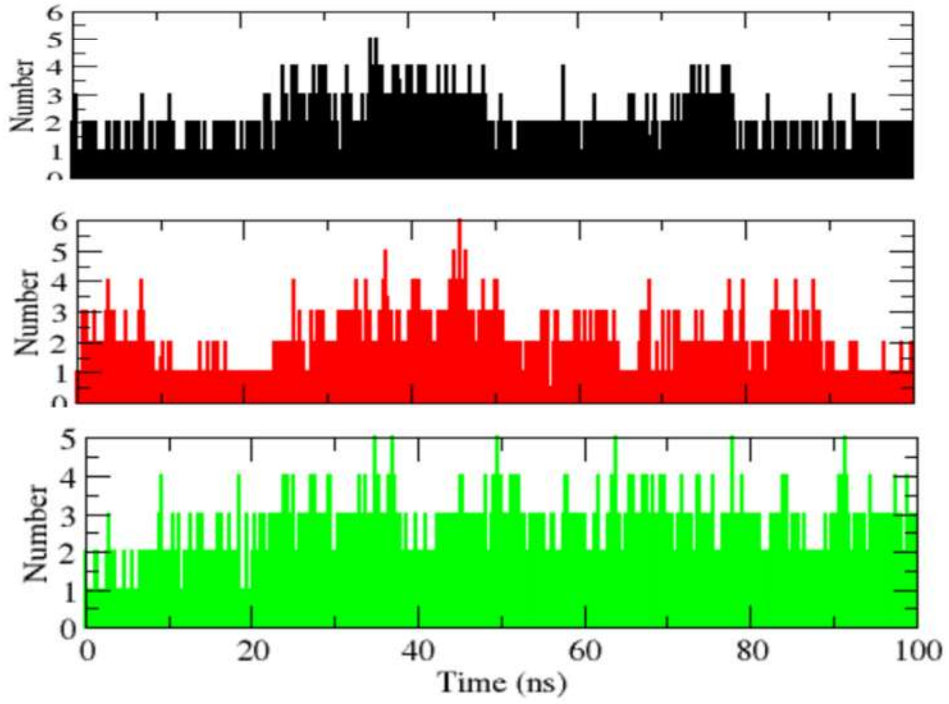

Number
Number
Number
Time (ns)
0
20
40
60
80
100

(B)

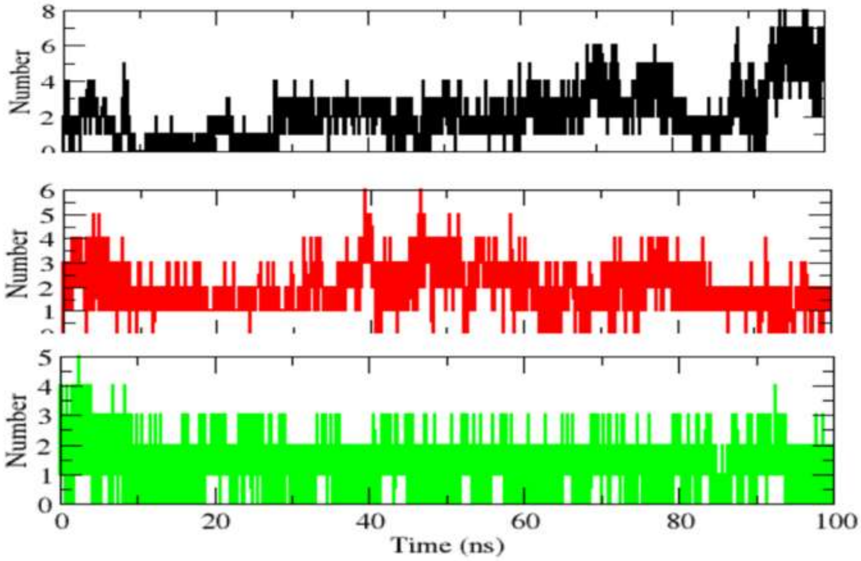

(C)

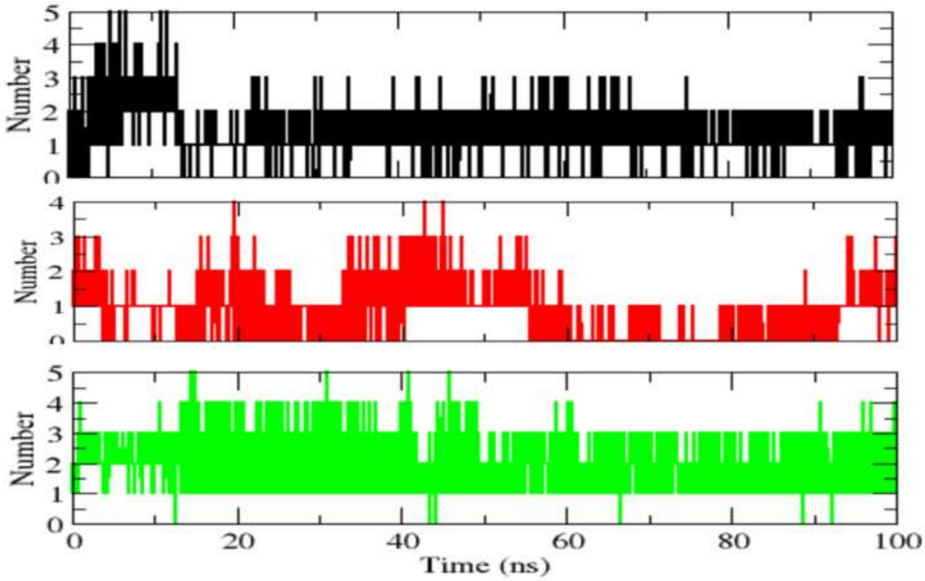

(D)

Figure S8. Hydrogen bonds analysis. The average number of hydrogen bonds between (A) Mrlip1-RHC80267, (B) Mrlip1- ZINC85530919, (C)Mrlip1- ZINC95914464 and (D) Mrlip1- ZINC85530320 as a function of time. The values were presented during 100,000 ps (100 ns) MD simulations time scale. The Black, Red and green colour represent the replica of complexes.

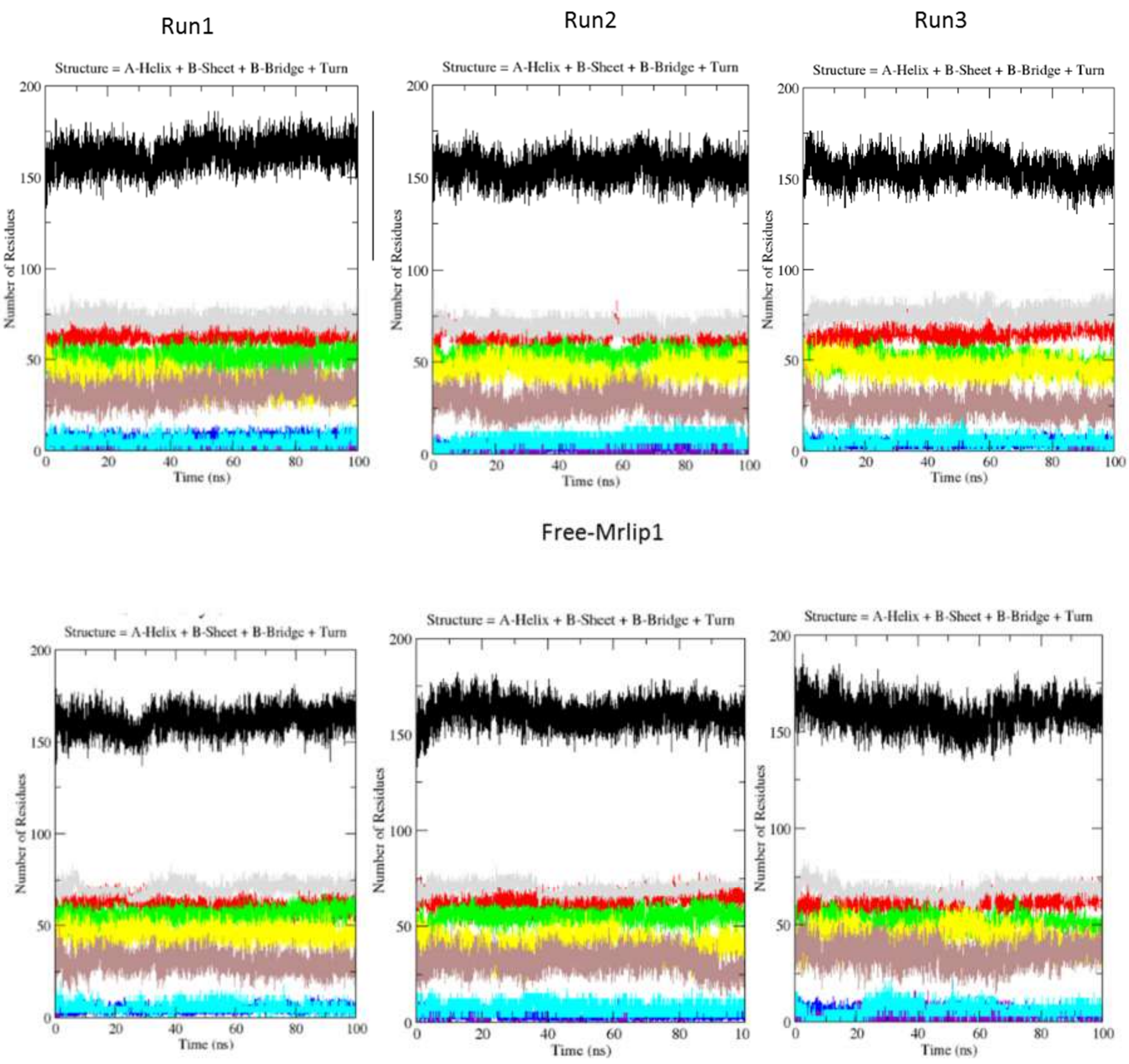

Mrlip1-RHC80267

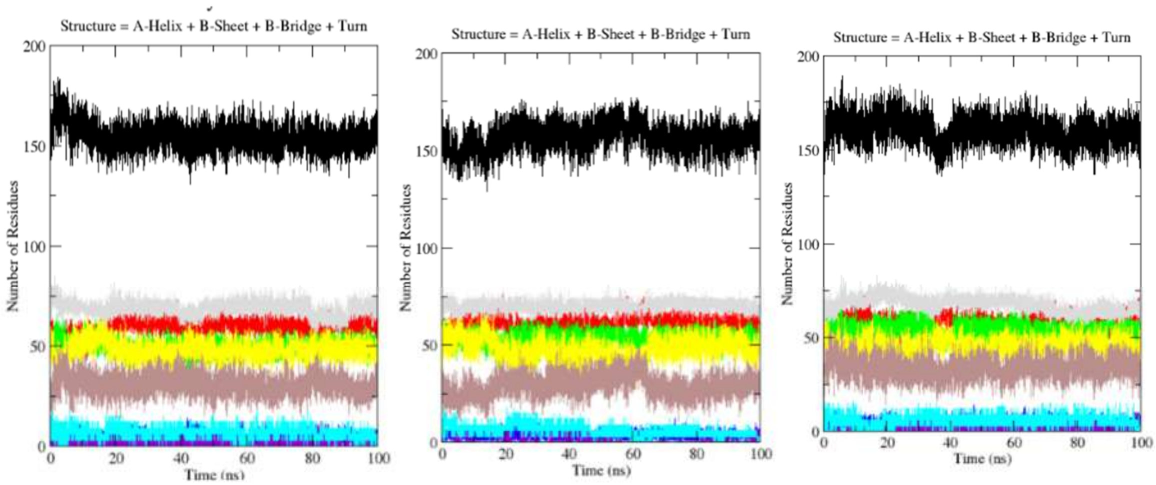

Mrlip1- ZINC85530919

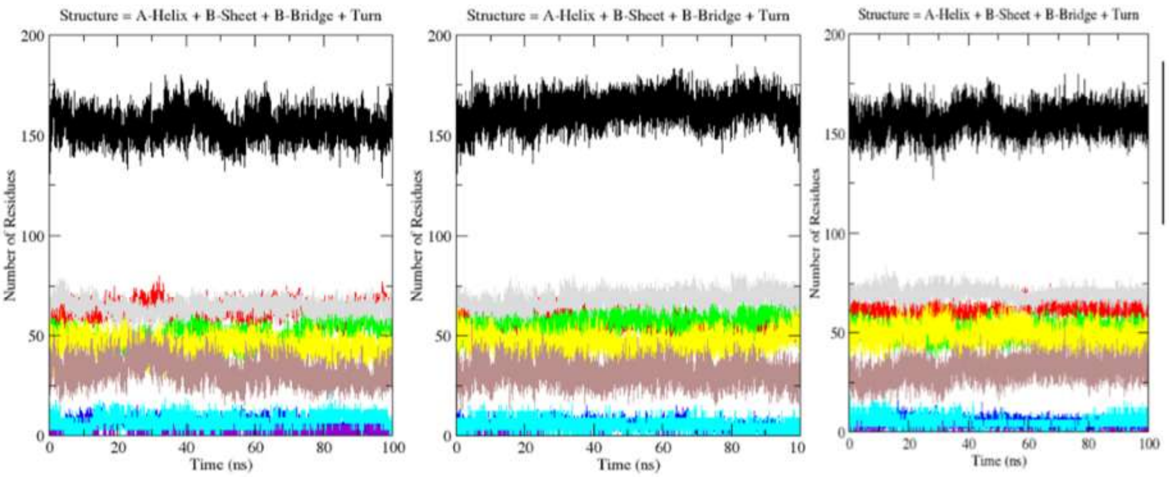

Mrlip1- ZINC95914464

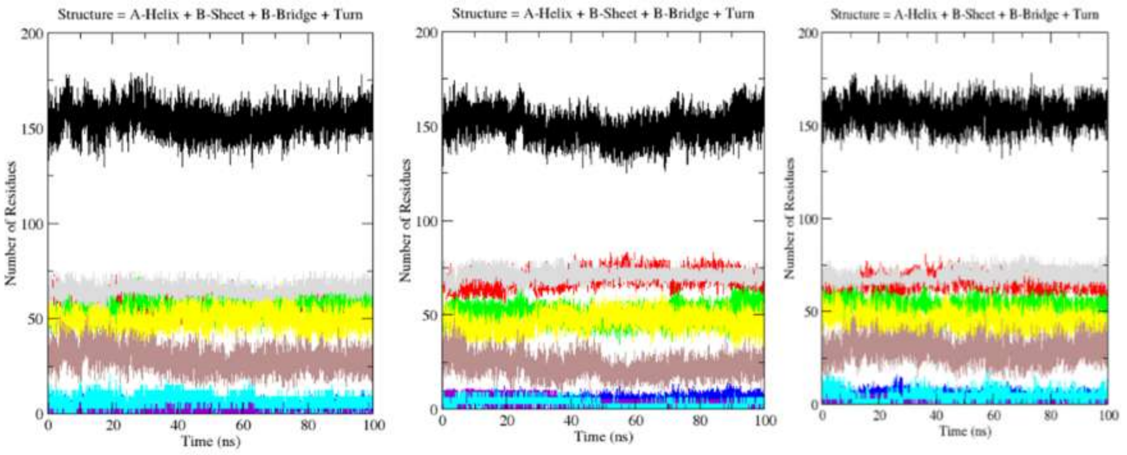

Mrlip1- ZINC85530320

Figure 9. Secondary structure plot. The secondary structure analysis indicating the structural elements present in **complexes** during the 100 ns MD simulations.

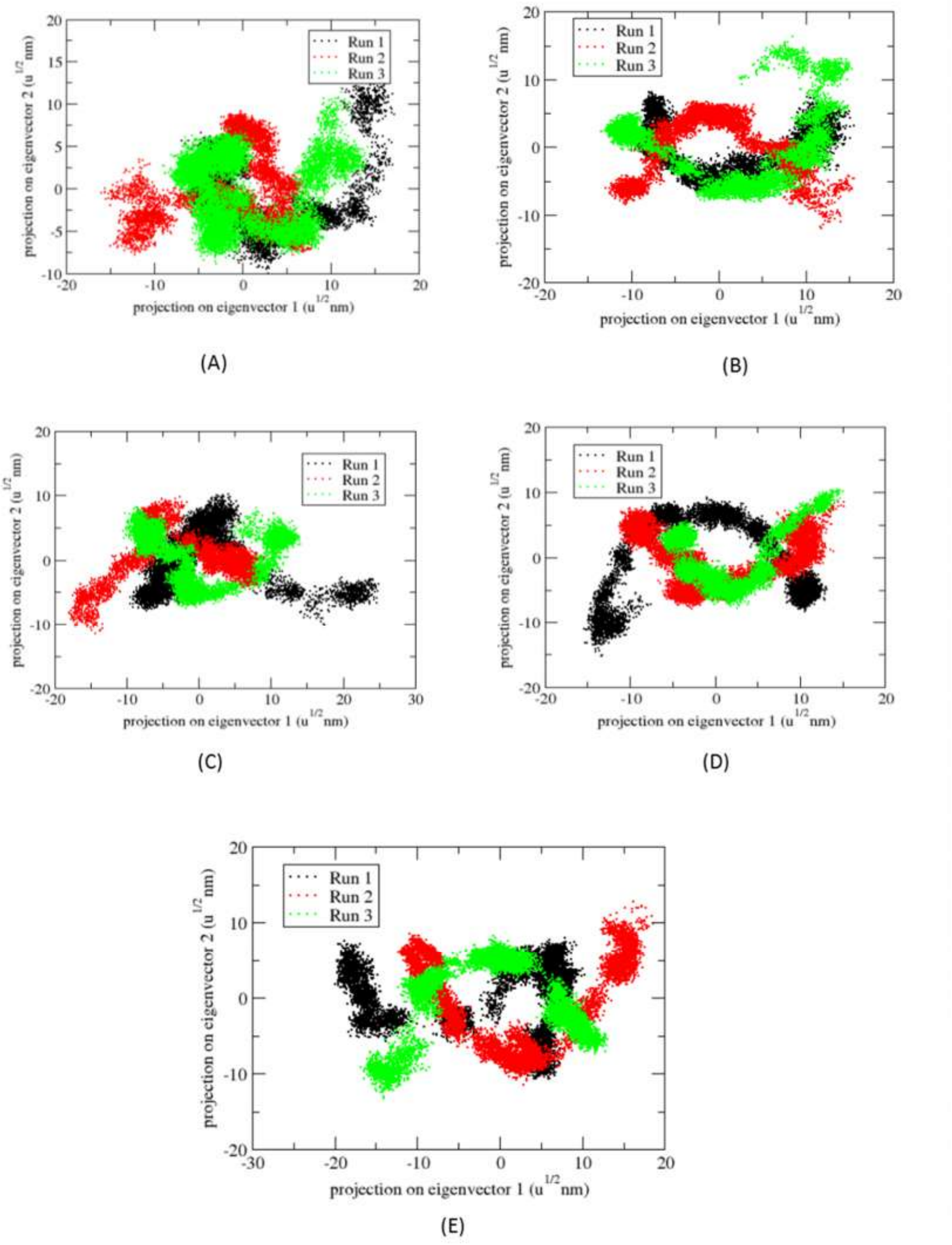

Figure S9. Principal Component Analysis. (A) The 2D projections of trajectories on eigenvectors showed different projections of Mrlip1. Black, red, and green colour represent values obtained from the different replica. (A) Free Mrlip1, (B) Mrlip1-RHC80267, (C) Mrlip1- ZINC85530919, (D) Mrlip1- ZINC95914464 and (E) Mrlip1- ZINC85530320.

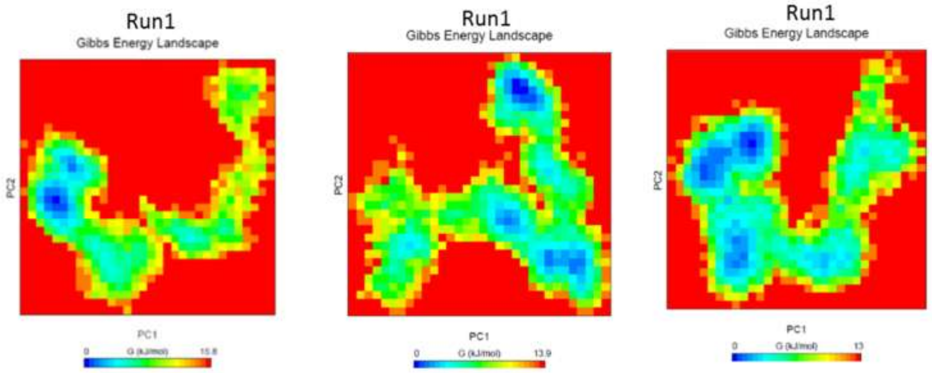

Free-Mrlip1

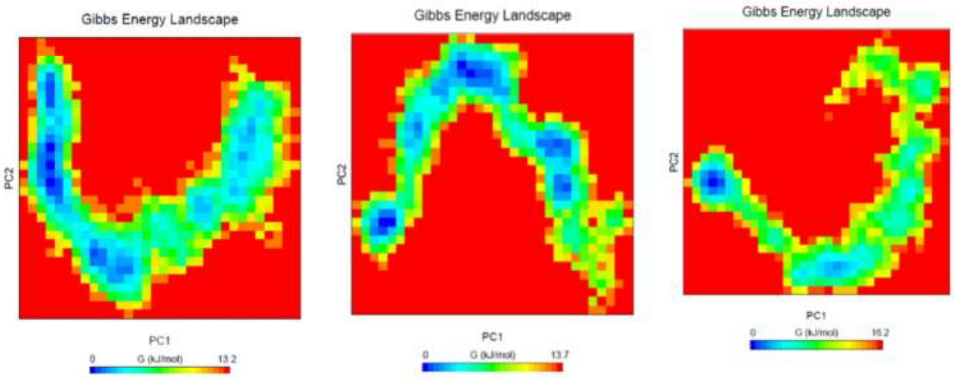

Mrlip1-RHC80267

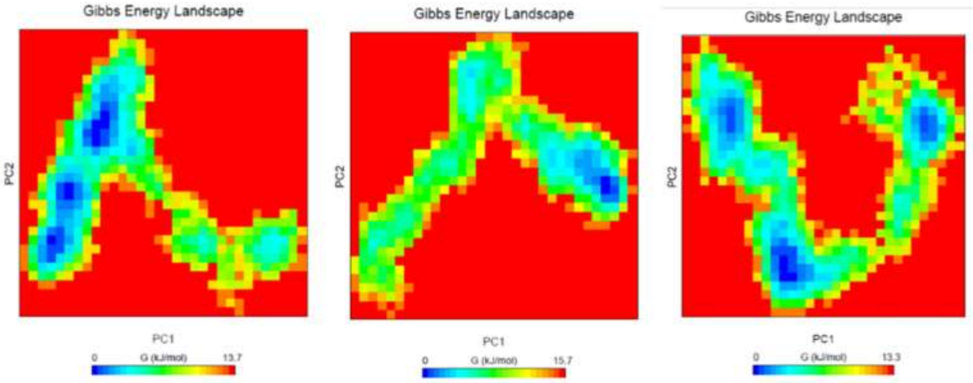

Mrlip1- ZINC85530919

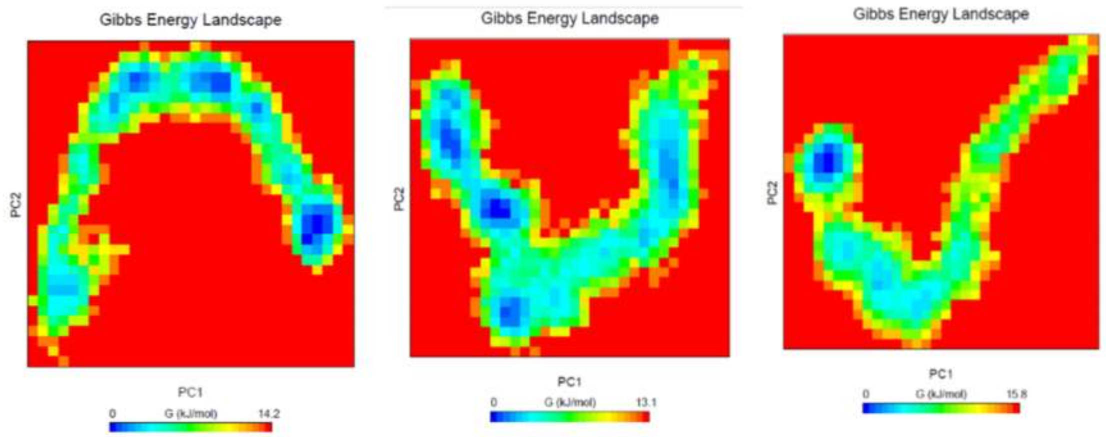

Mrlip1- ZINC95914464

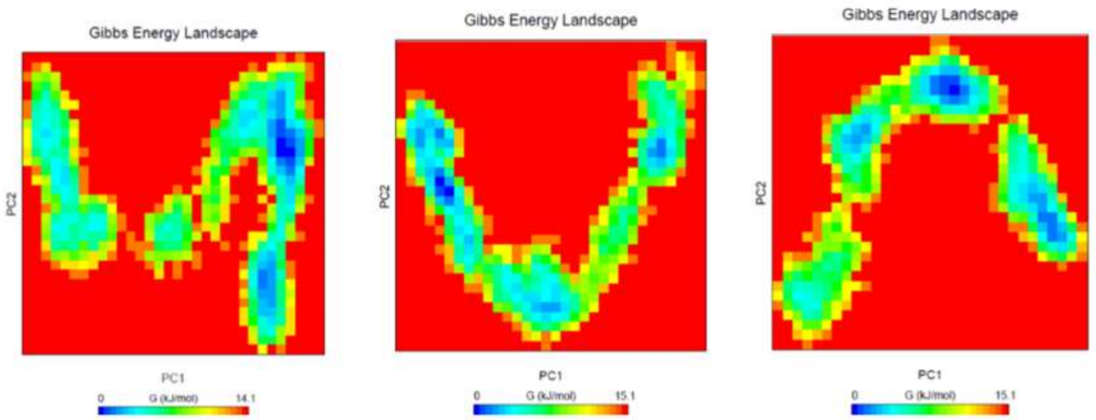

Mrlip1- ZINC85530320

Figure S10. The Gibbs energy landscape. The Gibbs energy landscape plot obtained during 100 ns MD simulations for Free Mrlip1, Mrlip1-RHC80267, Mrlip1-ZINC85530919, Mrlip1- ZINC95914464 and Mrlip1- ZINC85530320.
