# Supplementary material for: Identification and Evaluation of Inhibitors of Lipase from Malassezia restricta using Virtual High-Throughput Screening and Molecular Dynamics Studies
Source: Int J Mol Sci. 2019 Feb 18;20(4):884. doi: 10.3390/ijms20040884 (PMC6412828; doi:10.3390/ijms20040884)
Supplement: Supplementary file 1 [file ijms-20-00884-s001.zip › Supplementary_Table.pdf]

Table 1. List of top 80 compounds showing apparent binding affinity.

S. No.	Compound ID	Affinity (kcal/mol)	S. No.	Compound ID	Affinity (kcal/mol)	S. No.	Compound ID	Affinity (kcal/mol)	S. No.	Compound ID	Affinity (kcal/mol)
1.	RHC80267©	-6.5	21.	ZINC85542736	-10.7	41.	ZINC85569031	-10.7	61.	ZINC85593886	-10.6
2.	ZINC85491552	-11.2	22.	ZINC85542923	-10.6	42.	ZINC85569037	-10.6	62.	ZINC85593894	-10.8
3.	ZINC85530320	-10.6	23.	ZINC85543418	-11.5	43.	ZINC85569039	-10.7	63.	ZINC85593896	-10.7
4.	ZINC85530916	-10.9	24.	ZINC85542923	-10.6	44.	ZINC85569041	-10.8	64.	ZINC85593903	-10.6
5.	ZINC85530919	-10.7	25.	ZINC85543424	-11.0	45.	ZINC85569045	-11.1	65.	ZINC85593966	-10.8
6.	ZINC85531067	-10.7	26.	ZINC85543465	-10.7	46.	ZINC85569094	-10.9	66.	ZINC85593968	-10.8
7.	ZINC85531085	-11.0	27.	ZINC85543470	-11.9	47.	ZINC85569100	-10.8	67.	ZINC85626358	-10.7
8.	ZINC85531146	-11.1	28.	ZINC85543481	-10.7	48.	ZINC85569130	-10.7	68.	ZINC85627948	-10.7
9.	ZINC85531154	-11.2	29.	ZINC85545357	-11.3	49.	ZINC85569586	-10.6	69.	ZINC85628568	-10.7
10.	ZINC85531156	-12.8	30.	ZINC85545830	-11.1	50.	ZINC85570558	-10.6	70.	ZINC85628584	-10.8
11.	ZINC85531198	-11.3	31.	ZINC85545857	-10.6	51.	ZINC85570604	-11.1	71.	ZINC85628637	-10.7
12.	ZINC85531244	-10.8	32.	ZINC85546197	-11.2	52.	ZINC85570863	-10.6	72.	ZINC85632546	-11.0
13.	ZINC85531283	-12.0	33.	ZINC85546208	-10.9	53.	ZINC85570874	-10.9	73.	ZINC85632555	-10.6
14.	ZINC85531319	-11.0	34.	ZINC85546755	-10.6	54.	ZINC85593108	-10.7	74.	ZINC95910594	-12.9
15.	ZINC85531411	-10.8	35.	ZINC85546783	-10.7	55.	ZINC85593110	-11.2	75.	ZINC95911591	-10.8
16.	ZINC85531741	-11.4	36.	ZINC85568691	-10.7	56.	ZINC85593117	-10.9	76.	ZINC95911751	-10.6
17.	ZINC85542617	-11.7	37.	ZINC85569020	-11.3	57.	ZINC85593824	-10.6	77.	ZINC95914464	-11.2
18.	ZINC85542621	-11.7	38.	ZINC85569023	-11.7	58.	ZINC85593846	-10.5	78.	ZINC95914660	-11.7
19.	ZINC85542639	-12.0	39.	ZINC85569026	-11.6	59.	ZINC85593876	-10.6	79.	ZINC95914661	-10.8
20.	ZINC85542643	-10.7	40.	ZINC85569028	-11.9	60.	ZINC85593878	-11.2	80.	ZINC95919448	-11.5

© symbol denoted as control inhibitor compound.
